# Microgel culture and spatial identity mapping elucidate the signalling requirements for primate epiblast and amnion formation

**DOI:** 10.1242/dev.200263

**Published:** 2022-09-20

**Authors:** Clara Munger, Timo N. Kohler, Erin Slatery, Anna L. Ellermann, Sophie Bergmann, Christopher Penfold, Ioakeim Ampartzidis, Yutong Chen, Florian Hollfelder, Thorsten E. Boroviak

**Affiliations:** 1 Department of Physiology, Development and Neuroscience, University of Cambridge, Downing Site, Cambridge CB2 3EG, United Kingdom; 2 Centre for Trophoblast Research, University of Cambridge, Downing Site, Cambridge CB2 3EG, United Kingdom; 3 Wellcome Trust – Medical Research Council Stem Cell Institute, University of Cambridge, Jeffrey Cheah Biomedical Centre, Puddicombe Way, Cambridge CB2 0AW, United Kingdom; 4 Department of Biochemistry, University of Cambridge, Hopkins Building, Tennis Court Road, Cambridge CB2 1QW, United Kingdom; 5 Wellcome Trust – Cancer Research UK Gurdon Institute, Henry Wellcome Building of Cancer and Developmental Biology, University of Cambridge, Tennis Court Road, Cambridge, CB2 1QN, UK

## Abstract

The early specification and rapid growth of extraembryonic membranes are distinctive hallmarks of primate embryogenesis. These complex tasks are resolved through an intricate combination of signals controlling the induction of extraembryonic lineages and, at the same time, safeguarding the pluripotent epiblast. Here, we delineate the signals orchestrating primate epiblast and amnion identity. We encapsulated marmoset pluripotent stem cells into agarose microgels and identified culture conditions for the development of epiblast- and amnion-spheroids. Spatial identity mapping authenticated spheroids generated *in vitro* by comparison to marmoset embryos *in vivo*. We leveraged the microgel system to functionally interrogate the signalling environment of the postimplantation primate embryo. Single-cell profiling of the resulting spheroids demonstrated that ACTIVIN/NODAL signalling is required for embryonic lineage identity. BMP4 promoted amnion formation and maturation, which was counteracted by FGF-signalling. Our combination of microgel culture, single-cell profiling and spatial identity mapping provides a powerful approach to decipher the essential cues for embryonic and extraembryonic lineage formation in primate embryogenesis.

## Introduction

Mammalian life begins with concurrent establishment of embryonic and extraembryonic lineages. The zygote develops into a blastocyst consisting of the pluripotent epiblast, which gives rise to the embryo, and extraembryonic trophoblast and hypoblast, which form placenta and yolk sac, respectively. Upon implantation, the embryo undergoes major transformations (Bedzhov and Zernicka-Goetz, 2014). In human and non-human primate embryos, the epiblast polarises into a rosette and subsequently undergoes lumen expansion, thereby giving rise to the prospective amniotic cavity (Boroviak and Nichols, 2017; Ross and Boroviak, 2020; Rossant and Tam, 2018). Concomitantly, extraembryonic trophoblast cells mediate embryo attachment to the maternal endometrium. Inside the implanting conceptus, the hypoblast expands and diversifies into visceral endoderm covering the epiblast, parietal endoderm in the periphery and, in primates, is thought to generate extraembryonic mesoderm (Bianchi et al., 1993; Nakamura et al., 2016; Ross and Boroviak, 2020). The distal part of the epiblast rosette, in contact with the visceral endoderm, remains pluripotent, while the proximal part of the rosette, adjacent to trophoblast, differentiates into amnion (Enders et al., 1986; Rock and Hertig, 1948). Amnion cells downregulate the pluripotency factor *SOX2* and form a squamous epithelium, in contrast to the taller, columnar cells of the postimplantation epiblast (also *embryonic disc*) expressing the core pluripotency factors *NANOG*, *SOX*2 and *POU5F1* (also known as *OCT4*) (Hertig et al., 1956; Luckett, 1978). Nascent amnion and postimplantation epiblast present exceedingly similar global transcriptomes (Ma et al., 2019; Niu et al., 2019), which highlights the close kinship between embryonic and extraembryonic lineages in the primate embryo.

Lineage specification is orchestrated by a small suite of signalling pathways. In the early postimplantation embryo, the pluripotent epiblast compartment is exposed to BMP, FGF, WNT, and ACTIVIN/NODAL-ligands from surrounding extraembryonic tissues (Bergmann et al., 2022; Nakamura et al., 2016; Sasaki et al., 2016; Xiang et al., 2020). BMP-signalling has been implicated in amnion formation in postimplantation amniotic sac embryoids (Shao et al., 2017a; Shao et al., 2017b) and microfluidic-based models for amniogenesis (Zheng et al., 2019), but is also required for spatially organised germ layer and extraembryonic differentiation in human gastruloids (Chhabra et al., 2019; Deglincerti et al., 2016a; Tewary et al., 2017; Warmflash et al., 2014). Recent reports have shown that human epiblast cells readily differentiate into extraembryonic lineages *in vitro* (Dong et al., 2020; Linneberg-Agerholm et al., 2019; Rostovskaya et al., 2022; Xu et al., 2002; Zheng et al., 2019) and *in vivo* (Guo et al., 2021), suggesting greater lineage plasticity compared to rodent models (Chazaud and Yamanaka, 2016; Grabarek et al., 2012; Nichols and Gardner, 1984; Rossant, 2018). However, the signalling code required to protect epiblast identity in the primate embryo from precocious differentiation has remained elusive.

Human postimplantation development remains poorly understood because the relevant stages of *in vivo* developed embryos are difficult to access. Recent protocols for human blastocyst culture to postimplantation stages have opened exciting avenues to model embryo implantation in a dish (Deglincerti et al., 2016b; Shahbazi et al., 2016; Xiang et al., 2020), but human embryos are scarce and therefore not suitable for high-throughput functional interrogation. Stem cell-based embryo models are currently being developed to overcome these challenges (Chhabra et al., 2019; Harrison et al., 2017; Rivron et al., 2018; Shahbazi et al., 2017; Shao et al., 2017b; Shao et al., 2017a; Warmflash et al., 2014; Yu et al., 2021) and provide powerful alternatives for functional analysis of human embryogenesis (Moris et al., 2020; Shao et al., 2017a; Shao et al., 2017b; van den Brink et al., 2020; Zheng et al., 2019). Nevertheless, human models lack access to *in vivo* controls and, to date, no non-human primate stem cell-based embryo models have been developed to enable direct comparison to the postimplantation embryo by single-cell profiling within the same species.

To delineate the acquisition of regional lineage identities in primate embryogenesis *in vivo*, we recently conducted spatial embryo profiling in the common marmoset (Bergmann et al., 2022). Here, we encapsulate small populations of common marmoset pluripotent stem cells (cmPSCs) into agarose microgels. The scaffold provided by the soft gel creates a biomimetic 3D environment for the generation of epiblast (Epi)- and amnion (Am)-spheroids as a binary model for embryonic disc versus amnion lineage specification. Spatial identity mapping authenticated spheroids generated *in vitro* by comparison to marmoset embryos *in vivo*. We functionally assessed the complex signalling environment of the postimplantation primate embryo and show that the ACTIVIN/NODAL axis safeguards embryonic lineage identity, in contrast to FGF/MAPK and BMP signalling. Our work provides a paradigm to generate, interrogate and authenticate regionalised tissues of the primate embryo.

## Results

### Generation of primate Epi-spheroids in agarose microgels

**Fig. 1:**
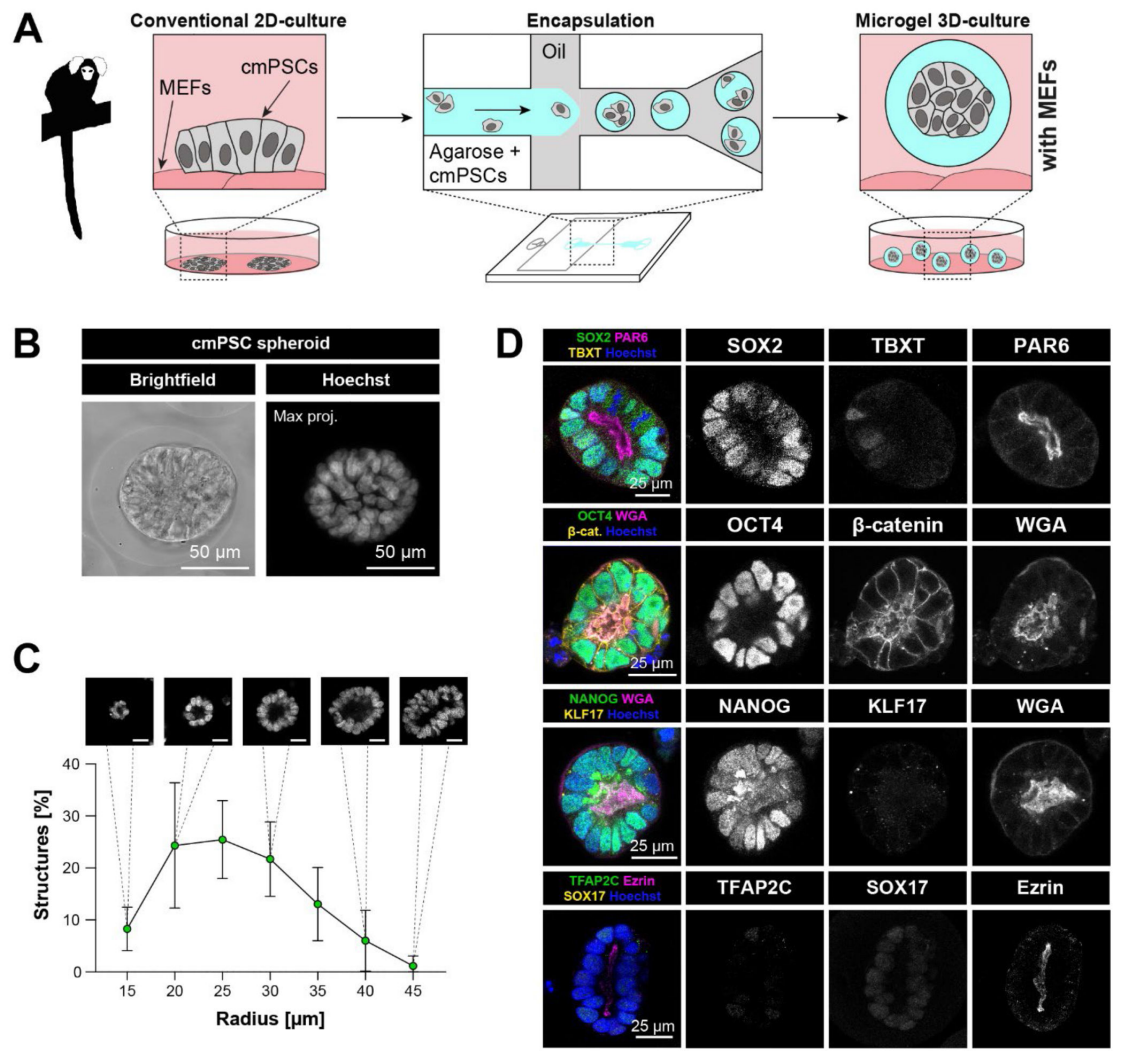
Encapsulation of cmPSCs in agarose microgels **A**, Workflow for encapsulation of cmPSCs into agarose microgels. **B**, Representative brightfield and confocal maximum projection (Hoechst) images of encapsulated primed cmPSCs cultured in N2B27 / 1% Matrigel at day 6. **C**, Size distribution of Epi-spheroids (N=5, 2 independent cell lines). Shapiro-Wilk normality test: p-value = 0.4138. Scale bar: 25 μm **D**, Confocal images of Epi-spheroids on day 6 generated from microgel encapsulated, primed cmPSCs in N2B27 supplemented with 1% Matrigel on MEFs.

We set out to establish a stem cell-based model in microgels to functionally interrogate marmoset embryogenesis. Naïve (Bergmann et al., 2022) and primed (Sasaki et al., 2005; Thomson et al., 1996) cmPSCs cultured in self-renewing conditions (PLAXA and KSR/FGF2, respectively) were dissociated and mixed with low melting agarose solution (Fig. 1A, Fig. S1). Monodisperse water-in-oil emulsion droplets were generated in microfluidic polydimethylsiloxane (PDMS) devices from two streams of aqueous and oil phases by break-off flow focussing (Kleine-Brüggeney et al., 2019; Schindler et al., 2021) to subsequently form the agarose microgels (Fig. 1A). The cmPSC-containing agarose microgels were transferred into conventional tissue culture plates containing mouse embryonic fibroblasts (MEFs) at the bottom of the dish.

We screened encapsulated cmPSCs in different experimental conditions to identify suitable culture regimes to model epiblast morphogenesis. Experimental conditions included media promoting self-renewal for naïve (PLAXA)(Bergmann et al., 2022) and primed (KSR/FGF2 and E8) pluripotency and differentiation permissive, serum-free N2B27. Furthermore, we examined the addition of either no extracellular matrix (ECM) or 1% Matrigel for all culture conditions (Fig. S1A). cmPSCs readily formed 3D-structures in agarose microgels, similar to human (h)PSCs (Schindler et al., 2021). 3D-structure formation of microgel encapsulated cmPSCs was more efficient in 1% Matrigel (40-80% of gels containing structures), than without ECM (15-40%) (Fig. S1B-E). To determine the cellular organisation and lineage identity of encapsulated marmoset 3D-structures, we performed whole-mount confocal immunofluorescence at day 6.

Naïve cmPSCs encapsulated in agarose microgels and cultured in PLAXA retained the naïve pluripotency factors KLF17 and TFAP2C and the core pluripotency factor SOX2, in line with preimplantation epiblast identity (Fig. S1B). Encapsulated naïve cmPSCs initiated polarity and rosette formation, but completely failed to undergo lumen expansion in PLAXA and at low frequency in primed or differentiating media (Fig. S1B,F). In differentiating media, loss of KLF17 expression in naïve cmPSCs correlated with lumen size (Fig. S1H). To determine the identity of KLF17-negative cells, we performed whole-mount immunofluorescence for pluripotency and differentiation markers after 6 days in N2B27 culture. The majority of cells retained expression of SOX2 and OCT4 and did not upregulate primitive streak (TBXT), endoderm (SOX17), mesoderm (PDGFRα), trophoblast (GATA3) or amnion (TFAP2A) markers, suggesting progression from naïve to primed pluripotency in microgel culture (Fig. S2).

Primed cmPSCs generated 3D-structures positive for SOX2, but negative for KLF17 and TFAP2C in differentiating and primed self-renewing conditions, similar to the marmoset (Bergmann et al., 2022), cynomolgus (Nakamura et al., 2016) and human (Tyser et al., 2020; Xiang et al., 2020) postimplantation epiblast (Fig. S1C). We observed upregulation of KLF17 and TFAP2C in encapsulated primed cmPSCs when cultured in PLAXA, indicating partial resetting towards naïve pluripotency (Fig. S1C). Primed cmPSCs formed polarised spheroids with a lumen in both self-renewing or differentiating conditions (Fig. 1B, Fig. S1C). 3D-structures in differentiating conditions (N2B27) exhibited the highest levels of lumen expansion (Fig. S1B,C,G). Collectively, these data are in accordance with the reported increase in lumenogenesis of human PSCs during the naïve to primed transition (Schindler et al., 2021; Shahbazi et al., 2017; Shao et al., 2017b).

Microgel cultures of primed cmPSCs in differentiation-permissive N2B27 formed homogenous spheroids, consisting of tall, columnar epithelial cells surrounding an expanded lumen (Fig. 1B-D, Fig. S1C) reminiscent of the embryonic disc in the early postimplantation embryo. Spheroid size followed a normal distribution in agarose microgels and with diameters of 50-70 μm (Fig. 1C). To determine the developmental identity of N2B27-cultured spheroids from primed cmPSCs, we performed confocal imaging with lineage markers of the early postimplantation embryo. Spheroids expressed core pluripotency factors SOX2, OCT4 and NANOG, apical polarity markers EZRIN, WGA and PAR6, low levels of TBXT (Brachyury) and lacked expression of the naïve pluripotency factor KLF17, amnion/trophoblast marker TFAP2C and the endoderm regulator SOX17 (Fig. 1D, Movie 1). This profile was consistent with postimplantation epiblast identity (Bergmann et al., 2022). Consequently, we refer to the 3D-structures derived from encapsulated primed cmPSCs in N2B27 on MEFs as Epi-spheroids.

### Amnion differentiation of encapsulated cmPSCs: Am-spheroids

**Fig. 2:**
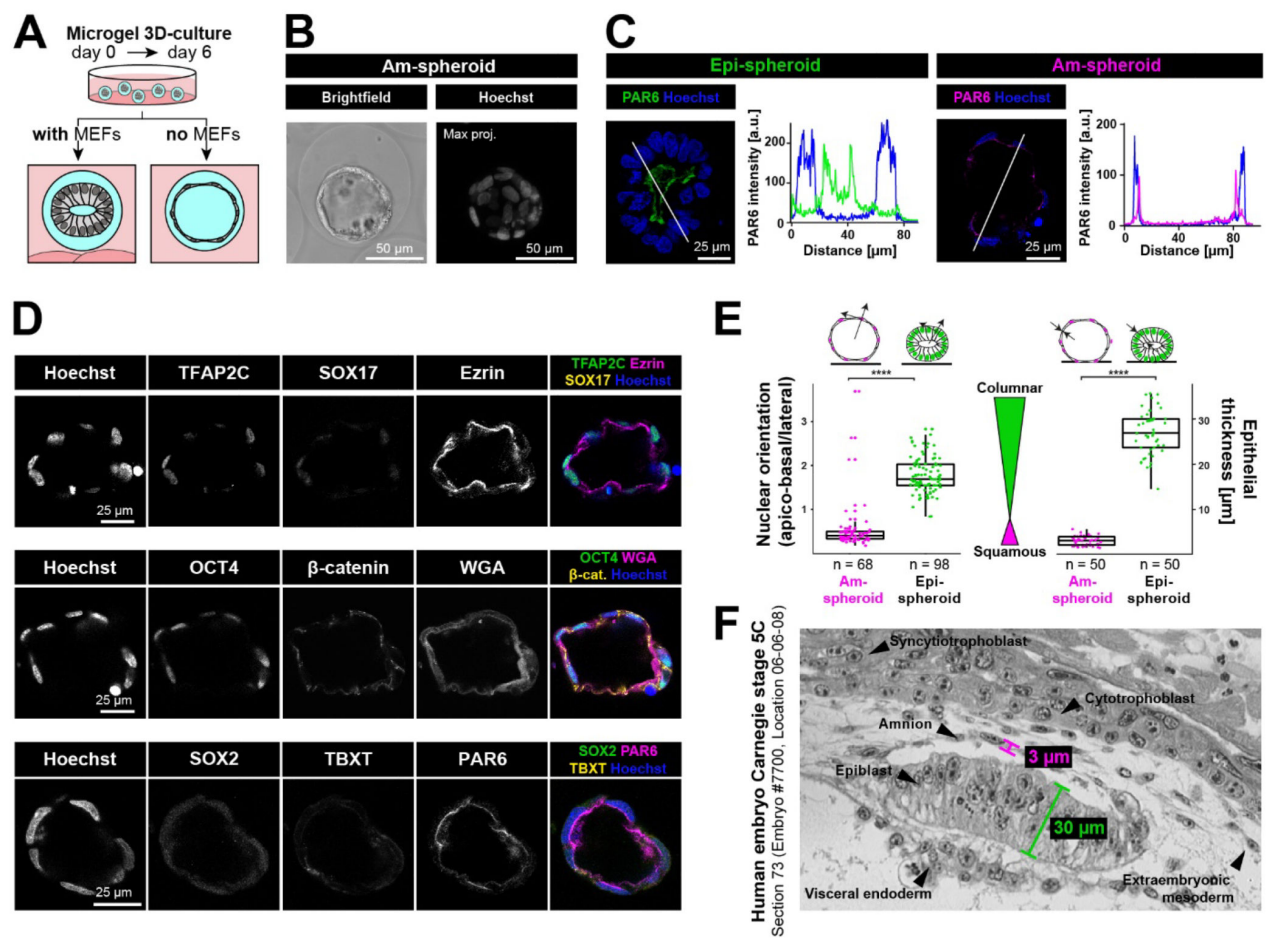
Generation of Am-spheroids **A**, Schematic illustration of microgel culture conditions testing removal of MEFs. **B**, Representative brightfield and confocal maximum projection (Hoechst) images of an Am-spheroid. **C**, Representative confocal immunofluorescence images of Epi-spheroids and Am-spheroids. Intensity profiles of pixels (shown on the right) are plotted along the white lines. **D**, Confocal immunofluorescence images of Am-spheroids at day 6. **E**, Box plot quantification of nuclear orientation and epithelial thickness (measured at cell junctions) of Am- and Epi-spheroids, n numbers indicated for each sample pooled from 2 independent experiments. Two-tailed Mann-Whitney test (****: p <0.0001). **F**, Histological cross-section of a human embryo at Carnegie stage 5C with epithelial thickness measurements of amnion and embryonic disc.

Microgel suspension culture of cmPSCs in the presence of MEFs showed sustained SOX2 expression across experimental conditions (Fig. S1C). To examine whether signals from MEFs at the bottom of the dish were required for epiblast identity, we removed MEFs from the N2B27-based culture regime (Fig. 2A). Strikingly, instead of undergoing Epi-spheroid formation, encapsulated cmPSCs developed into cysts consisting of a thin, squamous epithelium (Fig. 2B). Removal of MEFs did not lead to an increase in cell death (Fig. S3A,B). The cysts were polarised as indicated by the apical polarity markers EZRIN, WGA and PAR6 and expressed lineage markers OCT4 and TFAP2C, in the absence of SOX2, TBXT and SOX17 (Fig. 2C,D Fig. S3C,D, Movie 2). Loss of SOX2, together with TFAP2C and OCT4 expression is a distinguishing feature of nascent amnion in primates (Bergmann et al., 2022; Ma et al., 2019; Nakamura et al., 2016). Thus, we named the resulting structures Am-spheroids.

Am-spheroids underwent 8-fold larger lumen expansion than Epi-spheroids (Fig. 2C,E,F). Due to this expanded lumen size, Am-spheroids frequently outgrew the 100 μm agarose microgels (Fig. S3E). Escaped Am-spheroids could be collected and further grown in suspension culture (Fig. S3F). To capture differences in the cellular architecture between Epi- and Am-spheroids, we measured nuclear orientation and epithelial thickness. Nuclei in Epi-spheroids consistently pointed towards the centre of the lumen, in contrast to Am-spheroids, where nuclei were arranged in a perpendicular direction, pointing along the surface of the vesicle (Fig. 2E). Epithelial thickness was 10-fold increased in Epi- over Am-spheroids (Fig. 2E). Notably, the observed differences for nuclear orientation and epithelial thickness in the spheroid model were consistent with differences in the epithelial structure of human amnion and epiblast in the postimplantation embryo (Fig. 2F).

### Transcriptomic lineage mapping of Epi- and Am-spheroids to the primate embryo

**Fig. 3:**
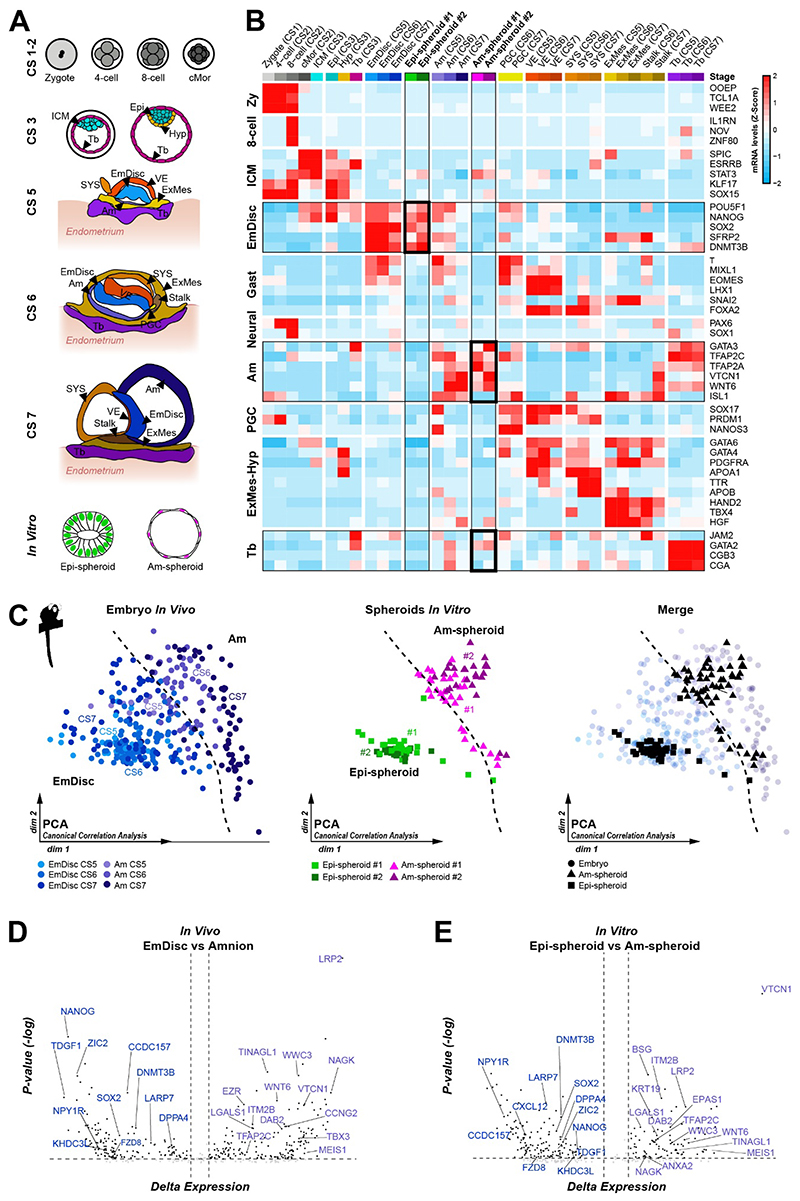
Single-cell profiling of Epi- and Am-spheroids for direct comparison to the marmoset postimplantation embryo **A**, Schematic illustration of all samples profiled in heatmap (b) including *in vivo* marmoset embryonic stages from Carnegie Stage (CS) 1 to CS 7 and *in vitro* Epi-spheroids and Am-spheroids. cMor: compacted morula, ICM: inner cell mass, Epi: epiblast, Hyp: hypoblast, Tb: trophoblast, EmDisc: embryonic disc, Am: amnion, VE: visceral endoderm, SYS: secondary yolk sac, ExMes: extraembryonic mesoderm, PGC: primordial germ cell. **B**, RNA expression heatmap of marmoset *in vivo* and *in vitro* samples including stage- and lineage-specific markers. For *in vitro* spheroids, z-score average expression based on 32 Epi-spheroids from cell line #1, 19 Epi-spheroids from cell line #2, 31 Am-spheroids from cell line #1 and 25 Am-spheroids from cell line #2. Zy: zygote, Gast: gastrulation. **C**, Principal component analysis of adjusted expression values using a set of genes identified via canonical correlation analysis. Displayed are the embryonic disc (CS 5-7), amnion (CS 5-7) and Epi- and Am-spheroids. Segregation between EmDisc and Am samples is highlighted with a support vector machine generated decision boundary. **D,E**, Differentially expressed genes in the (**D**) EmDisc *vs.* amnion (*in vivo*) and (**E**) Epi- *vs.* Am-spheroids (*in vitro*).

To assess the developmental identity of Epi- and Am-spheroids by comparison to the marmoset embryo, we isolated spheroids from the microgels through Agarase treatment (Fig. S3G) and performed full-length single-cell transcriptome profiling (Picelli et al., 2014). The embryo dataset (Bergmann et al., 2022) included preimplantation lineages from Carnegie stages (CS)1-3 and postimplantation stages CS5, CS6 and CS7 (Fig. 3A). Global transcriptome comparison demonstrated that Epi-spheroids exhibited highest similarity with the early embryonic disc (CS5 and CS6) (Fig. S4A). Epi-spheroids expressed core pluripotency and postimplantation epiblast lineage markers, in the absence of differentiation- or preimplantation-associated genes (Fig. 3B, Fig. S4C).

Am-spheroids showed close correspondence to CS6 amnion and expressed the amnion markers *TFAP2A*, *TFAP2C*, *VTCN1* and *WNT6* (Fig. 3B, Fig. S4A). Importantly, Am-spheroids did not express the trophoblast-specific transcripts *JAM2*, *CGA*, or *CGB3* (Fig. 3B). We observed downregulation of embryonic disc-associated factors, including *SOX2*, *NANOG*, *SFRP2* and *DNMT3B*, but also *ISL1*, a gene heterogeneously expressed in the marmoset amnion and gastrulating cells *in vivo* (Bergmann et al., 2022) (Fig. S4C). Integrated analysis showed that amnion and epiblast lineage separated along the first and second principal component (Fig. 3C). We computed a support-vector-machine-decision boundary based on *in vivo* samples to facilitate Epi- and Am-spheroid mapping (Fig. 3C, S4C). Am-spheroids robustly clustered with CS6 amnion, and Epi-spheroids grouped with *SOX2*-positive postimplantation epiblast samples at across CS5-7 (Fig. S4C). To independently examine whether Epi- and Am-spheroids differentiate along the embryonic disc and amnion lineage, we compared differentially expressed genes between embryonic disc and amnion at CS6 (Fig. 3D) and Epi- and Am-spheroids (Fig. 3E). We observed a broad overlap of lineage-specific transcripts (Fig. 3D,E, Fig. S4C) and gene ontology (Fig. S4B). These observations demonstrate that Epi- and Am-spheroids recapitulate the cellular architecture, polarity and transcriptome-wide signature of CS6 embryonic disc and amnion, respectively, and validate microgel cultures as a biomimetic maquette to investigate primate development.

### ACTIVIN/NODAL signalling is critical for epiblast identity

**Fig. 4:**
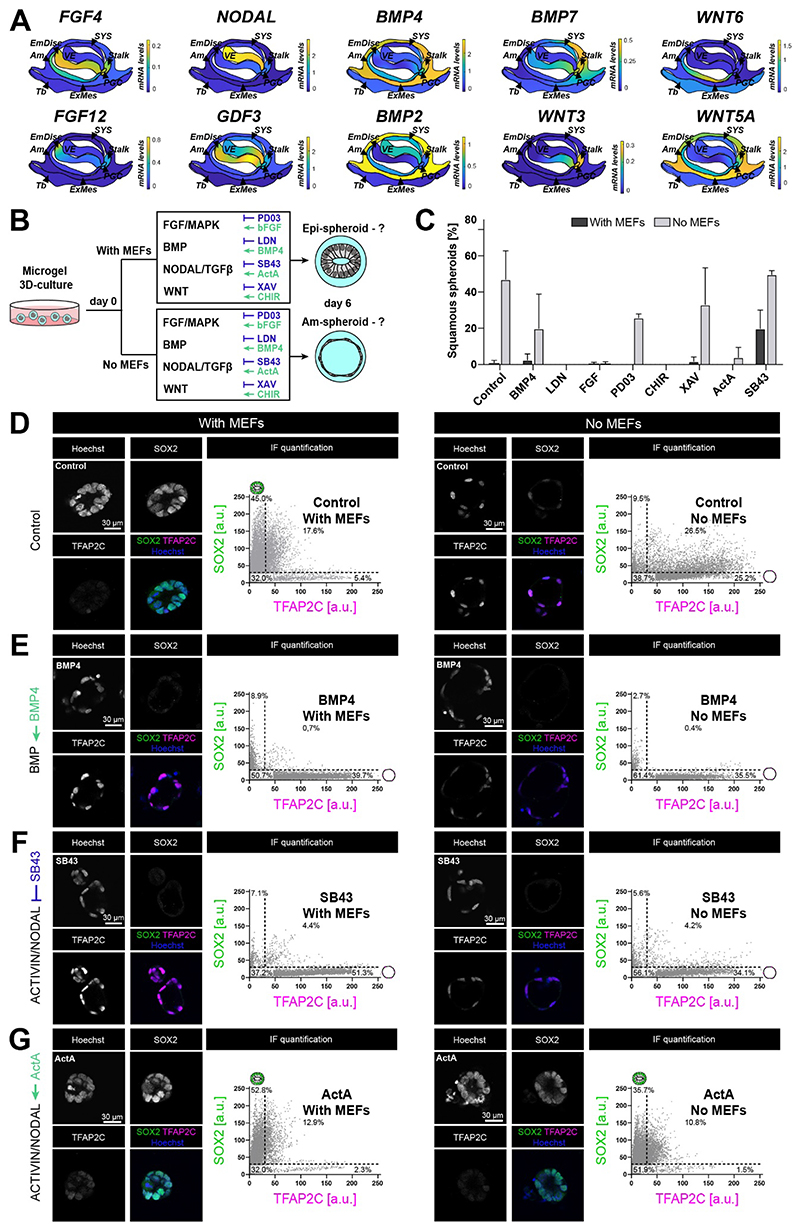
Single pathway perturbation delineates the essential signals for Epi- and Am-spheroid formation **A**, Spatial transcriptome data of the CS6 postimplantation marmoset embryo (Bergmann *et al.*, 2021). **B**, Screening conditions used to probe signalling pathways that promote or inhibit Epi- and Am-spheroid fate. **C**, Squamous spheroid forming capacity of microgel cultured primed cmPSCs (N=2, two independent cell lines) at day 6 under tested conditions. **D-G**, Confocal immunofluorescence images of control Epi- and Am-spheroids (**D**), when treated with BMP4 (**E**), SB43 (**F**), or ActA (**G**). Cells were stained for SOX2 (EmDisc) and TFAP2C (amnion). Fluorescence intensity (shown on the right) was quantified on Hoechst masks for each frame in the z-stack.

Spatial transcriptome profiling in the early postimplantation marmoset embryo revealed the signalling environment in space and time, featuring multiple ligands of the BMP, FGF/MAPK, WNT and ACTIVIN/NODAL pathways (Bergmann et al., 2022) (Fig. 4A). We reasoned that the microgel suspension culture regime, generating Epi-spheroids in the presence of MEFs and Am-spheroids in the absence of MEFs, provides a powerful platform to functionally dissect the signals required for embryonic disc and amnion formation. Single-cell RNA-seq of MEFs used for microgel suspension culture demonstrated expression of ligands from FGF/MAPK, BMP, ACTIVIN/NODAL and WNT signalling cascades (Fig. S5A). To delineate the individual roles of BMP, FGF/MAPK, WNT and ACTIVIN/NODAL signalling, we activated or inhibited each signalling pathway during Epi- and Am-spheroid formation (Fig. 4B). Consistent with previous results, control conditions gave rise to Epi-spheroids in the presence of MEFs and Am-spheroids without MEFs (Fig. 4C, Fig. S5B). Encapsulated cmPSCs displayed robust cell survival throughout culture conditions, apart from BMP inhibition with LDN193189 (LDN) (Fig. S5C,D). BMP4 induced amnion both with and without MEFs, in accordance with observations in human postimplantation amniotic sac embryoids (Shao et al., 2017b; Shao et al., 2017a)(Fig. 4C, Fig. S5C). Activation of FGF/MAPK signalling with FGF2 was compatible with Epi-spheroid morphology, but ablated Am-spheroid development (Fig. 4C, Fig. S5C). Interestingly, MEK inhibition with PD0325901 (PD03) did not change Epi- or Am-spheroids formation compared to controls (Fig. 4C, S5C). WNT activation with the glycogen synthase kinase (GSK3β) inhibitor CHIR99021 (CHIR) shifted development towards embryoid body-like structures without lumen (Fig. 4C, S5C). Inhibition of WNT signalling did not alter the dynamics of postimplantation epiblast and amnion formation in the microgel suspension culture system, similar to PD03 (Fig. 4C, S5C). In contrast, inhibition of the ACTIVIN/NODAL signalling pathway with SB431542 (SB43) resulted in Am-spheroid formation in the presence of MEFs, i.e. reversing cell fates compared to control conditions. Remarkably, activation of ACTIVIN/NODAL with ActivinA (ActA) had an equally profound effect leading to the formation of Epi-spheroids in the absence of MEFs (Fig. 4C, S5C). This suggests that epiblast versus amnion formation is predominantly controlled by the ACTIVIN/NODAL axis in the microgel culture system.

To capture the dynamics of lineage allocation, we performed whole-mount confocal imaging for all experimental conditions with the early lineage markers SOX2 (embryonic disc) and TFAP2C (amnion) (Fig. 4D-G). We determined nuclear SOX2 and TFAP2C intensity levels on each individual frame and plotted SOX2/TFAP2C ratios in scatterplots as a quantitative readout for Epi- and Am-spheroids formation (Fig. 4D-G, Fig. S6, S7). Untreated controls cultured in the presence of MEFs resulted in SOX2-positive Epi-spheroids with a columnar epithelium; in the absence of MEFs, encapsulated cmPSCs predominantly gave rise to TFAP2C-positive Am-spheroids (Fig. 4D). Microgel culture in the presence of FGF2 increased SOX2 levels in the absence of MEFs, but led to inconsistent columnar epithelial architecture across the individual structures (Fig. S6A). In the primate embryo, visceral endoderm expresses insulin growth factor 1 (IGF1) (Bergmann et al., 2022), which effectively supports the human pluripotency network *in vitro* (Wamaitha et al., 2020). We tested IGF1 in the Epi- and Am-spheroid assays and observed slightly decreased efficiencies for amnion formation, similar to FGF2 (Fig. S6F). PD03 exerted only modest effects on epiblast or amnion specification (Fig. 4C, Fig. S6A). Activation of WNT signalling resulted in loss of both SOX2 and TFAP2C, while WNT inhibition had a limited effect on Epi- and Am-spheroid formation (Fig. S6B). BMP activation shifted almost the entire population towards TFAP2C positive amnion with and without MEFs (Fig. 4E). BMP inhibition through LDN was toxic in the absence of MEFs, but not in the presence of MEFs (Fig S5D, S7A), potentially suggesting that BMP is required for amnion lineage entry. We repeated this experiment using Noggin, a soluble BMP-inhibitor. In contrast to LDN, Noggin was not toxic for spheroid formation in the absence of MEF and exhibited only a mild decrease in appearance of squamous spheroids (Fig. S7B). Microgels cultured with Noggin in the absence of MEFs expressed slightly elevated levels of SOX2 and lower levels of TFAP2C, compared to Am-spheroid controls (Fig. S7C-E). Thus, BMP inhibition with Noggin slowed down loss of epiblast identity, but did not prevent Am-spheroid formation.

Modulation of the ACTIVIN/NODAL signalling pathway profoundly changed cell fates in microgel suspension culture, with SB43 shifting the balance towards TFAP2C-positive Am-spheroids with and without MEFs (Fig. 4F). Notably, SB43 treatment with MEFs (Epi-spheroid condition) yielded more homogenous TFAP2C-positive Am-spheroids (Fig. 4F) than control Am-spheroids in the absence of MEFs (Fig. 4D). Positive stimulation of the ACTIVIN/NODAL axis with ActA robustly blocked TFAP2C expression and induced SOX2 levels in the absence of MEFs (Fig. 4G). Transforming growth factor beta (TGFβ), commonly used in self-renewing human PSC culture, upregulated SOX2, but did not block TFAP2C induction in the absence of MEFs (Fig. S6C-E). We conclude that Epi-spheroid formation was compatible with FGF/MAPK or WNT inhibition, but critically depended on ACTIVIN/NODAL signalling.

### Amnion lineage entry is regulated by ACTIVIN/NODAL, BMP and FGF

**Figure 5:**
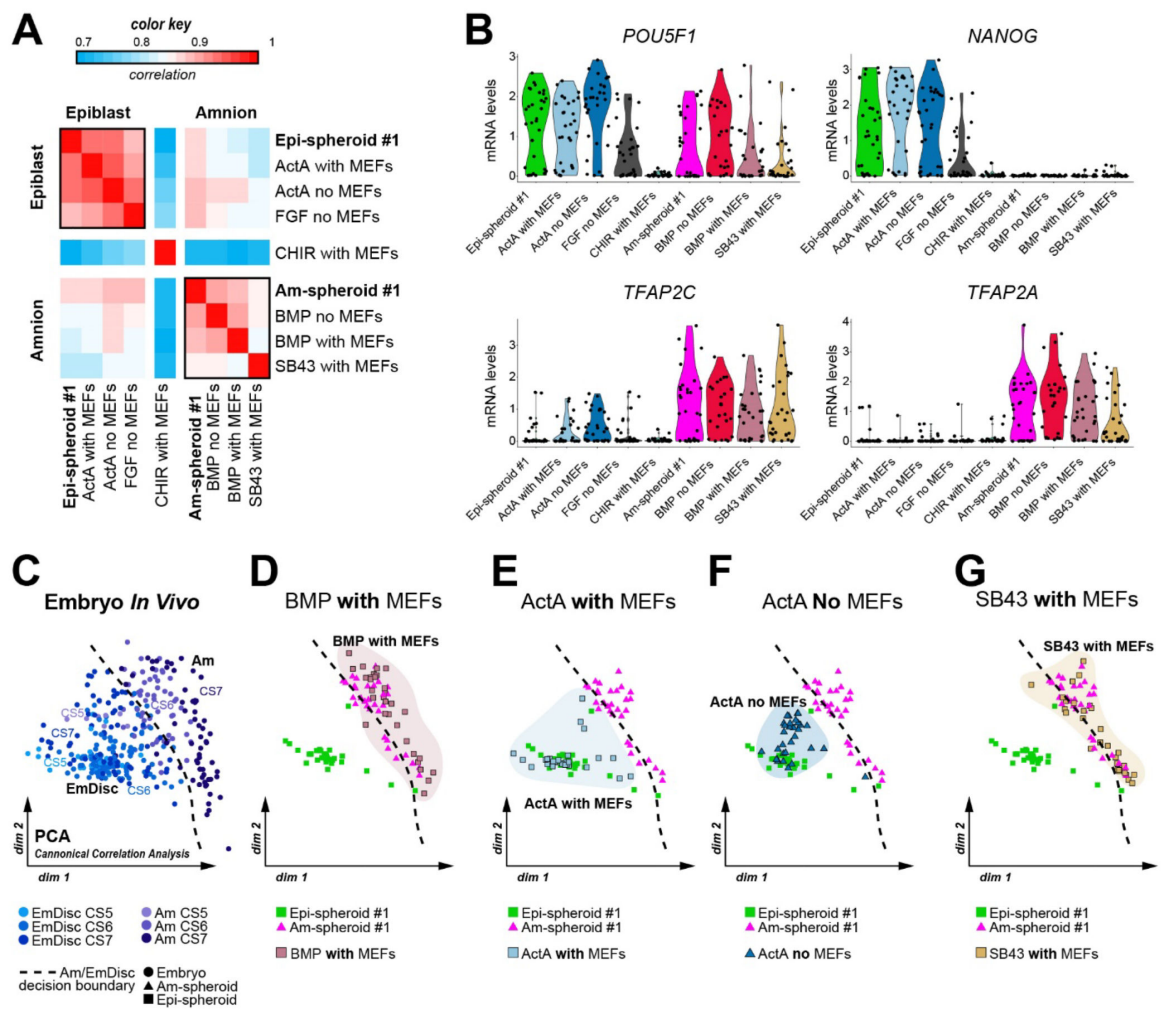
Genome-wide transcriptional assessment of lineage converted Epi- and Am-spheroids **A**, Pearson correlation analysis of control Epi-, Am-spheroids and specific pathway perturbations for lineage conversion conducted in cell line #1 with or without MEFs. Correlation is pseudo-bulk of all cells passing quality control (Epi-spheroid #1: 32 cells, ActA with MEFs: 29 cells, ActA no MEFs: 31 cells, FGF2 no MEFs: 31 cells, CHIR with MEFs: Am-spheroid #1: 31 cells, 31 cells, BMP4 no MEFs: 25 cells, BMP4 with MEFs: 31 cells, SB43 with MEFs: 30 cells) **B**, Single-cell RNA-seq Seurat-normalized mRNA levels for *POU5F1* (EmDisc & amnion lineage marker), *MIXL1* (Gastrulation marker), *TFAP2C* and *TFAP2A* (amnion lineage markers). **C-G**, Principal component analysis of adjusted expression values using a set of genes identified via canonical correlation analysis comparing *in vivo* dataset to control Epi- and Am-spheroids (**C**) with the perturbed samples: BMP4 with MEFs (**D**) Activin-A with (**E**) and without (**F**) MEFs, and SB43 with MEFs (**G**). Silhouettes and decision boundary respectively highlight the perturbation shown in each plot and the segregation of EmDisc and amnion samples.

To examine the implications of signalling pathway modulation for embryo lineage entry, we performed single-cell transcriptome profiling of ActA, SB43, CHIR, FGF2 and BMP4-treated spheroid cultures. Transcriptome-wide correlation analysis showed that ActA-treated cultures with and without MEFs and FGF2-stimulated spheroids correlated with Epi-spheroids (Fig. 5A). In contrast, BMP4-treated spheroids with and without MEFs and microgel cultures with SB43 on MEFs resembled Am-spheroids (Fig. 5A). These lineage-converted spheroids upregulated epiblast and amnion markers (Fig. 5B), in the absence of neural (*SOX1*), trophoblast (*CGB3*) or hypoblast (*TTR, SOX17*) associated gene expression, including extraembryonic mesoderm (*HGF*) (Fig. S8A). We individually assessed perturbation experiments with embryonic disc and amnion samples from the postimplantation marmoset embryo (CS5-7). BMP4 conditions with or without MEFs promoted amnion lineage identity (Fig. 5C,D, Fig. S8B). FGF stimulation resulted in a substantial shift towards embryonic disc in spheroid culture without MEFs, but localised close to the decision boundary (Fig. S8B). CHIR treatment led to upregulation of *FOXF1, HAND1*, *CDX2* and *SNAI2*, consistent with advanced mesodermal fate (Tyser et al., 2021) (Fig. S8D,F). ActA treatment of spheroids with or without MEFs overlapped with embryonic disc identity (Fig. 5E,F), although ActA without MEFs upregulated *TBXT* and MIXL1 (Fig. S8A). Finally, SB43-induced reversal of embryonic disc to amnion fate in spheroid cultures with MEFs resulted in complete overlap with control Am-spheroids and amnion samples from the marmoset embryo (Fig. 5G). These results demonstrate that amnion specification occurs in the absence of ACTIVIN/NODAL or through induction of BMP signalling, with a subordinate role for FGF/MAPK.

### FGF signalling counteracts amnion formation

**Figure 6:**
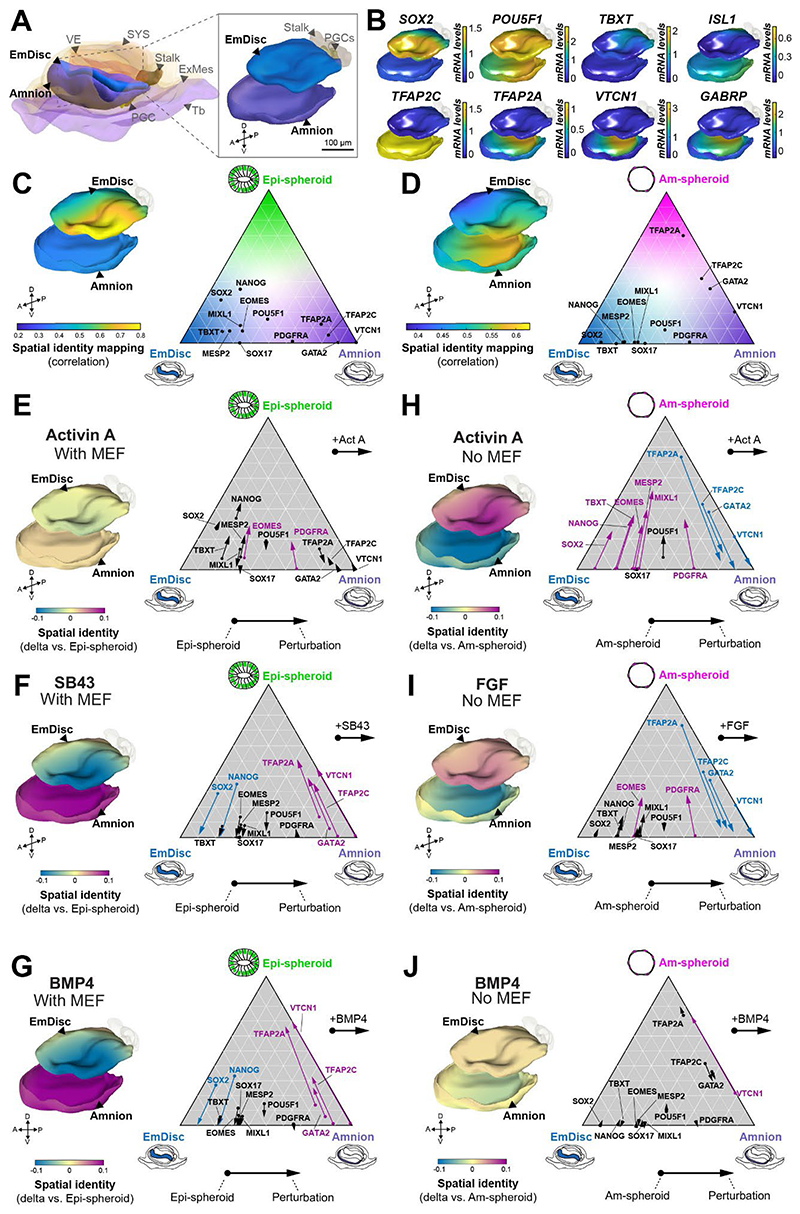
Dynamic spatial identity mapping of Epi- and Am-spheroid cultures **A**, 3D reconstruction of the CS6 marmoset embryo (Bergmann *et al.*, 2021) highlighting the EmDisc and amnion. Additional lineages are displayed in low opacity for context. **B**, Representative gene expression patterns depicted in Gaussian process regression-based 3D models of CS6 EmDisc and amnion. Expression patterns demarcate anterior EmDisc,posterior EmDisc, and spatial domains of the amnion. **C-D**, Comparison of Epi-spheroids (**C**) and Am-spheroids (**D**) to the embryo through static spatial identity mapping and comparative ternary plots. Spatial identity mapping colour scale represents projection of correlation values onto embryo model surfaces followed by Gaussian process regression mapping. Ternary plots display proportional mRNA levels of lineage markers given by distance to the vertex. **E-J**, Dynamic spatial identity and comparative ternary plots for indicated screen conditions with MEF (**E-G**) or without MEF (**H-J**). In ternary plots, arrow origin denotes levels in control spheroids, and arrow endpoint denotes levels when indicated signalling pathway perturbation is applied.

We assessed spheroids obtained from the single pathway perturbation assay by dynamic spatial identity mapping to delineate the influence of each signalling pathway on the regional identity of the embryo. Global transcriptome comparison indicated highest similarity of spheroids to embryonic stage CS6 (Fig. S4A), thus we focused spatial identity mapping on CS6 embryonic disc and amnion (Fig. 6A). Core pluripotency factors *SOX2* and *POU5F1* are expressed in the anterior embryonic disc, with *TBXT* demarcating the posterior compartment (Bergmann et al., 2022) (Fig. 6B). Amnion broadly expresses the lineage markers *TFAP2A* and *TFAP2C*, while *VTCN1* and *GABRP* are confined towards central regions (Bergmann et al., 2022) (Fig. 6B). *ISL1* is expressed in the primitive streak and at low, heterogenous levels throughout the amnion (Fig. 6B). We performed spatial identity mapping of Epi- and Am-spheroids by projecting a similarity metric based on correlation onto embryo samples followed by Gaussian process regression (Bergmann et al., 2022) to interpolate values to the entire embryo surface. Epi-spheroids showed highest correlations with anterior embryonic disc regions (Fig. 6C). In contrast, Am-spheroids were most similar to mature amnion regions (Fig. 6D). For *dynamic* spatial identity mapping, we plotted the changes in lineage identity on the virtual embryo model in comparison to the static correlations for Epi- (Fig. 6C) and Am-spheroids (Fig. 6D). We monitored lineage markers in ternary plots next to dynamic spatial identity graphs to examine the effects of individual signalling perturbations (Fig. 6E-J). Dynamic spatial identity mapping highlights the embryonic region undergoing changes in lineage identity, hence ActA treatment of encapsulated cmPSCs with MEFs, i.e. Epi-spheroid conditions, did not cause changes (Fig. 6E). However, SB43 (Fig. 6F) and BMP4 (Fig. 6G) on MEFs caused a dramatic shift towards amnion. ActA without MEFs, i.e. Am-spheroid conditions, effectively changed regional identity towards the anterior embryonic disc, however, samples also mapped towards a transitional zone between embryonic disc and amnion to a similar degree (Fig.6G). Activation of FGF/MAPK signalling directed lineage identity away from mature amnion towards the embryonic disc (Fig. 6I). FGF signalling effectively suppressed amnion lineage markers, but only marginally upregulated pluripotency-associated transcripts (Fig. 5B,6I,S8C). Thus, the FGF/MAPK axis counteracts acquisition of the transcriptional circuitry of the amnion, rather than inducing embryonic disc identity.

### BMP and ACTIVIN/NODAL coordinate amnion maturation

**Figure 7:**
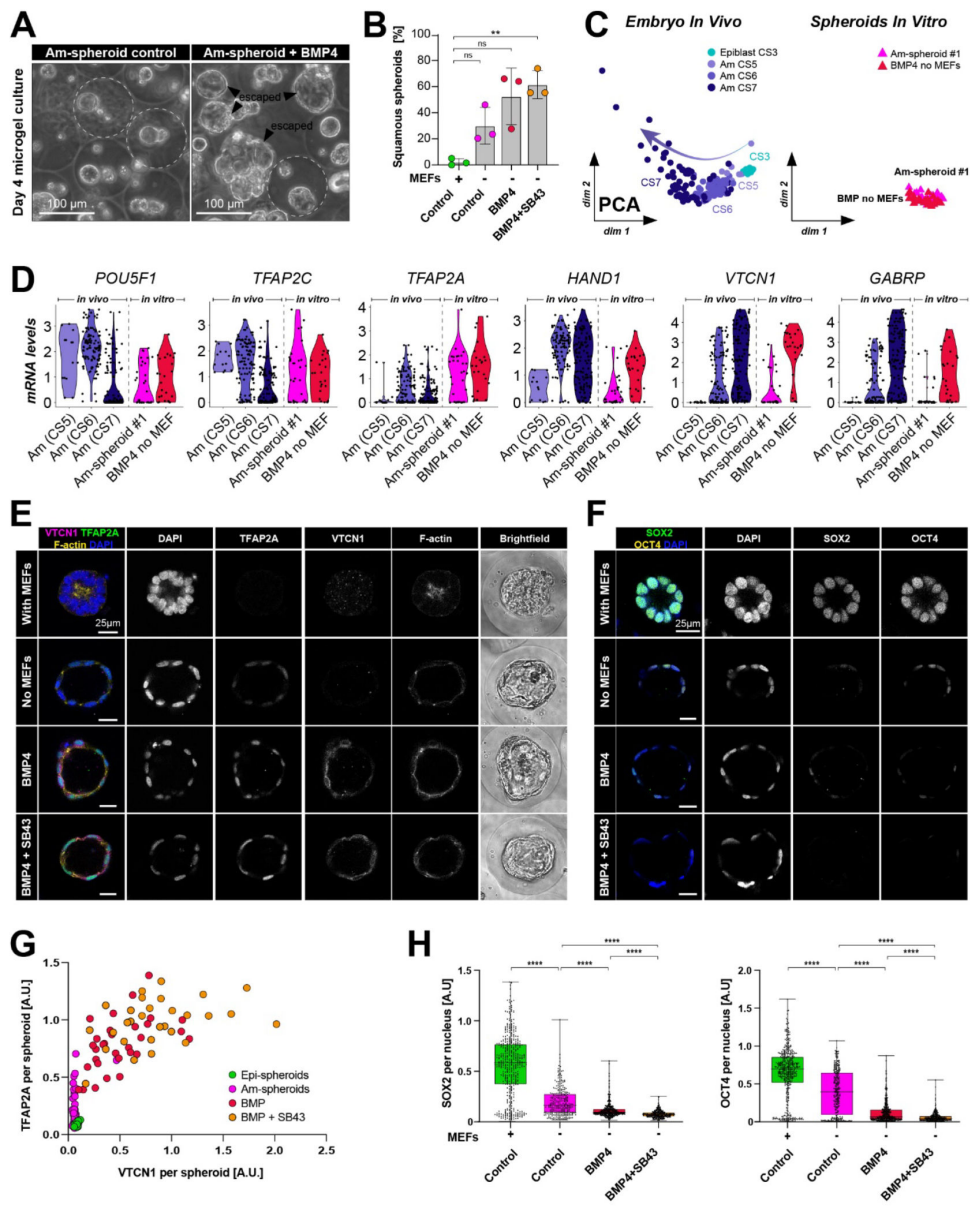
BMP coordinates amnion maturation *in vitro* **A**, Brightfield images of control and BMP4 treated Am-spheroids on day 4 of culture. Encapsulated Am-spheroids highlighted by white circular outlines and escaped Am-spheroids indicated with black arrows. **B**, Squamous spheroid forming capacity of control Am-spheroids and Am-spheroids treated with BMP4 or BMP4 and SB43 (N=3). Two-tailed t-test with Welch’s correction (Control with MEF *vs*. Control no MEF: p=0.071, Control with MEF vs. BMP4: p=0.0545, Control with MEF vs. BMP4+SB43: p=0.008). **C**, Principal component analysis of 10,000 most variable genes comparing control and BMP4 treated Am-spheroid with the *in vivo* amnion (CS5-7) and preimplantation epiblast (Epiblast CS3) for developmental context (PC1: 7.1%, PC2: 6.6%). **D**, Violin plots of mature amnion marker mRNA levels (Seurat normalised levels) of *in vivo* amnion (CS5-7) compared to control and BMP4 treated Am-spheroids. **E**-**F**, Confocal immunofluorescence images of control Am- and Epi-spheroid and of Am-spheroids under the indicated conditions at day 6. **G**, Quantification of fluorescence intensity for VTCN1 and TFAP2A measured per spheroid from the sum of the slices of z-stacks. **H**, Quantification of fluorescence intensity per nuclei normalised to DAPI. Kruskal-Wallis followed by Dunn’s multiple comparisons test (****: p <0.0001).

We noticed that Am-spheroids cultured in the presence BMP4 exhibited faster growth dynamics than control Am-spheroids (Fig. 7A). BMP4 induced large squamous spheroids that escaped from the agarose microgels by day 4 and quantification of Am-spheroids at day 3 showed that BMP accelerated Am-spheroid formation (Fig. 7B). To assess whether this acceleration led to developmentally more advanced stage, we compared the BMP4-treated and control Am-spheroids to amnion *in vivo*. PCA analysis with CS3 epiblast and CS5-7 amnion samples revealed that BMP4-treated spheroids clustered slightly towards the more mature CS7 amnion (Fig. 7C), which was accompanied by upregulation of mature amnion markers *HAND1*, *VTCN1* and *GABRP* (Fig 7D). However, BMP4 treatment was not sufficient for downregulation of *POU5F1,* indicating only partial maturation (Fig 7D). Considering that ActA promoted Epi-spheroid identity, we tested whether BMP activation together with ACTIVIN/NODAL inhibition further promotes amnion maturation by extinguishing residual pluripotency-associated transcripts. Similar to BMP4, combined BMP4 and SB43 treatment increased the formation of squamous epithelial spheroids at day 3 (Fig. 7B). This combinatorial approach resulted in Am-spheroids with particularly thin nuclei, reflected in a higher basolateral/apical nuclear orientation than BMP4 treatment alone or control Am- or Epi-spheroids (Fig. S9A-D). Lineage marker quantification showed that the mature amnion marker VTCN1 was highly expressed in both BMP4 and BMP4+SB43 treatments (Fig. 7E,G, Fig. S9D), in agreement with transcriptional upregulation (Fig. 7D). TFAP2A was also upregulated in both BMP4 and BMP4+SB43 cultures and correlated with VTCN1 levels (Fig. 7E,G). Strikingly, BMP4+SB43 treatment induced downregulation of OCT4 and completely suppressed SOX2 expression at the protein level (Fig. 7F,H). Thus, BMP4 both promotes amniogenesis and supports amnion maturation, which can be further accelerated through ACTIVIN/NODAL inhibition.

## Discussion

**Figure 8:**
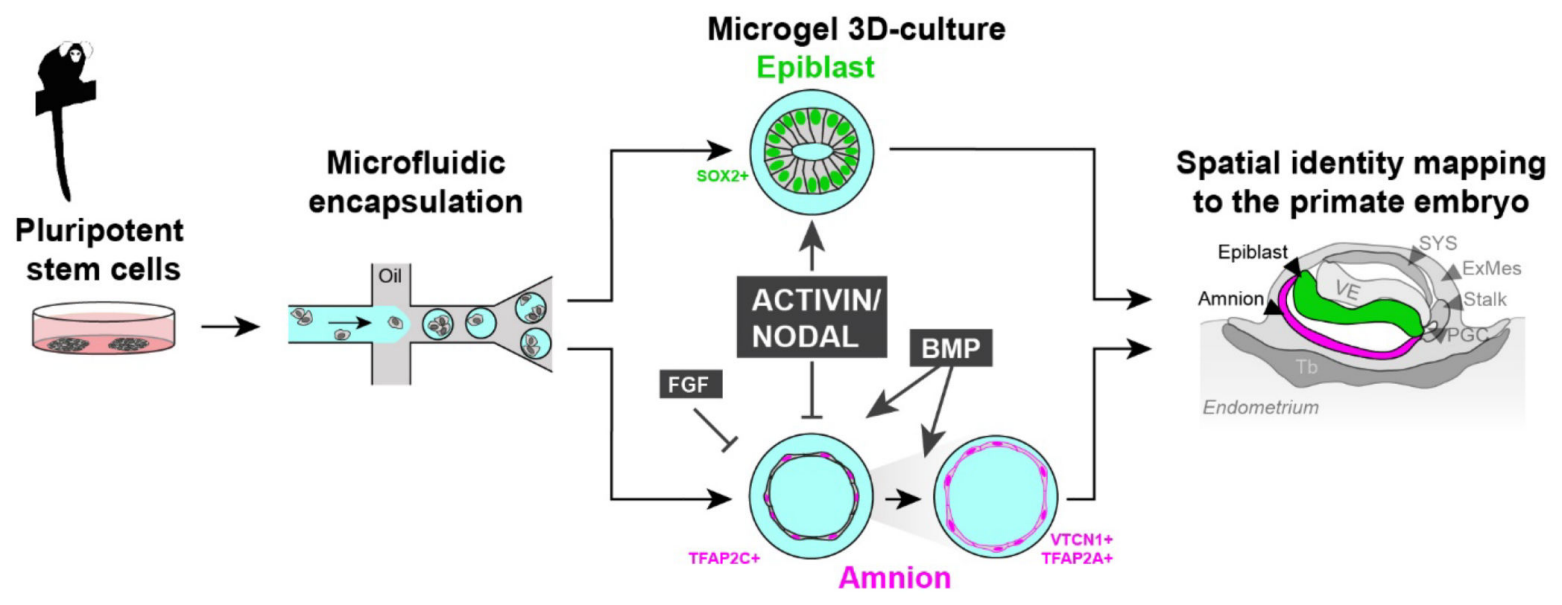
Schematic illustration of *in vitro* primate amniogenesis. In 3D-microgel culture of cmPSCs, ACTIVIN/NODAL axis safeguards embryonic lineage identity, in contrast to FGF/MAPK and BMP signalling. BMP also drives amnion maturation toward a POU5F1-negative, VTCN1- and TFAP2A-positive state.

Morphological and transcriptional analysis suggest that marmoset spheroid cultures represent a bona fide 3D-culture system to emulate epiblast and amnion formation *in vitro*. Epi-spheroids consisted of a columnar epithelium, in contrast to Am-spheroids which comprise thin, squamous tissue surrounding an expanded lumen. Recent models for human amniogenesis include the generation of amnion-like tissue in amnion-on-a-chip (Zhu et al., 2020) microfluidic devices (Zheng et al., 2019) and conventional 2D tissue culture (Guo et al., 2021; Rostovskaya et al., 2022). Importantly, working with marmoset cells enabled direct authentication of lineage identity by comparison to our spatial embryo profiling dataset (Bergmann et al., 2022). This approach allowed us to dissect and quantitatively assess the individual roles of the major signalling cascades controlling epiblast and amnion specification (Fig. 8).

Single pathway perturbation assays in spheroid cultures revealed that ACTIVIN/NODAL was the only pathway capable of reversing the epiblast versus amnion lineage decision. This result highlights a predominant role of ACTIVIN/NODAL for embryonic lineage identity. *NODAL* is expressed in the primate preimplantation epiblast, in contrast to mouse (Blakeley et al., 2015; Boroviak et al., 2018; Nakamura et al., 2016; Petropoulos et al., 2016; Yan et al., 2013) and, upon implantation, visceral endoderm becomes the major source of *NODAL* in human, cynomolgus and marmoset embryos (Bergmann et al., 2022; Ma et al., 2019; Nakamura et al., 2016). Inhibition of ACTIVIN/NODAL diverts human naïve pluripotent cells towards trophoblast (Guo et al., 2021), in accordance with the signalling requirements for human trophoblast stem cells, which include ACTIVIN/NODAL inhibitors SB43 and A83-01 as critical components (Okae et al., 2018; Turco et al., 2018). Although the ACTIVIN/NODAL pathway is not essential for establishing pluripotency in the embryo (Boroviak et al., 2015), it is required for long-term maintenance of naïve cultures in the marmoset (Bergmann et al., 2022). This is consistent with human naïve culture conditions, which require ACTIVIN/NODAL signalling for maintenance of the naïve pluripotency gene regulatory network (Guo et al., 2021; Osnato et al., 2021) and frequently either use MEFs or include ACTIVIN/NODAL ligands (Takashima et al., 2014; Theunissen et al., 2014). In primed human PSCs corresponding to the postimplantation epiblast (Bergmann et al., 2022; Nakamura et al., 2016), ACTIVIN/NODAL suppresses neuronal differentiation and is essential for the maintenance of the pluripotency circuitry (Beattie et al., 2005; Vallier et al., 2009; Xu et al., 2008; Zorzan et al., 2020). However, this function is transformed by WNT ligands during gastrulation to induce germ layer formation (Yoney et al., 2018; Yoney et al., 2021). We have identified localised expression of WNT3 and WNT8A in the posterior embryonic disc of the marmoset embryo, together with continuous, high level expression of *NODAL* from the adjacent visceral endoderm (Bergmann et al., 2022). Considering the pronounced effects of ActA and SB43 in the marmoset spheroid culture assay, we propose a model where ACTIVIN/NODAL signalling preserves pluripotency in the anterior compartment of the embryonic disc (Bergmann et al., 2022). This safeguarding mechanism is likely required to prevent both extraembryonic and neuronal differentiation prior to gastrulation and to sustain pluripotency until somitogenesis.

BMP signalling promoted amnion formation, consistent with previous stem cell-based embryo models in human (Shao et al., 2017a; Shao et al., 2017b; Zheng et al., 2019). In the marmoset embryo, the visceral endoderm broadly expresses the BMP inhibitors *CHRD* and *NOG*, probably to confine BMP-induced amnion formation towards the periphery of the embryonic disc (Bergmann et al., 2022). Notably, previous *in vitro* studies interrogated the effects of exogenous signals on lineage specification exclusively in the presence of FGF-ligands (Yoney et al., 2018; Zheng et al., 2019). In contrast, we examine the relevant signalling pathway individually and show that FGF/MAPK primarily blocks amnion lineage acquisition, rather than actively supporting embryonic disc identity. This may suggest a role for FGF in pacing amnion formation.

Rodent embryogenesis is orchestrated by strict lineage bifurcation events between embryonic and extraembryonic tissues (Chazaud and Yamanaka, 2016; Nichols and Smith, 2009; Rossant and Tam, 2009). Conversion of mouse naïve pluripotent stem cells into trophoblast or hypoblast requires either genetic manipulation (McDonald et al., 2014; Niwa et al., 2000; Schrö et al., 2015; Wang et al., 2011) or prolonged differentiation (Cho et al., 2012). In contrast, human and non-human primate naïve PSCs are prone to spontaneous differentiation into extraembryonic lineages, including trophoblast, hypoblast and amnion (Dong et al., 2020; Guo et al., 2021; Linneberg-Agerholm et al., 2019; Shao et al., 2017b; Theunissen and Jaenisch, 2014). This increased developmental plasticity might be a consequence of primate-specific features of early embryogenesis, in particular the rapid growth of extraembryonic membranes. Primate embryos specify amnion, produce substantial amounts of extraembryonic mesenchyme and develop a primary and secondary yolk sac prior to gastrulation (Boroviak and Nichols, 2017; Ross and Boroviak, 2020). The requirement to generate such lineage diversity early on is likely to confer a higher regulative capacity for cells of the primate embryo. At the same time, mechanisms must be in place to protect the embryonic lineage from precocious differentiation. Our results suggest that ACTIVIN/NODAL signalling from the visceral endoderm protects the embryonic lineage in the epiblast and thus provides a developmental rationale for the role of ACTIVIN/NODAL and FGF signalling in promoting human and non-human primate PSC self-renewal.

Microgel encapsulation of human and non-human primate PSCs establishes a powerful technical foundation to disentangle the signals controlling embryogenesis and to address comparative evolutionary questions about extraembryonic lineage specification. Encapsulated primed cmPSCs readily formed Am-spheroids, whereas human PSCs in the same conditions gave rise to embryonic disc-like structures (Schindler et al., 2021). At the same time, primed cmPSCs transcriptionally correspond to CS5/6, which is slightly earlier than primed human PSCs (Bergmann et al., 2022; Nakamura et al., 2016). It is tempting to speculate that amnion formation may be leveraged as a functional readout for CS5/CS6-equivalent PSCs in human. Alternatively, human PSCs may require higher levels of BMP-signalling and further studies will be required. With a throughput exceeding 100 microgels per second, very large numbers of cell-laden biomimetic microspheres can be generated, providing easy access to defined primate embryo compartments that can be authenticated by direct comparison to the embryo. Microgel encapsulation can be readily extended to incorporate extraembryonic tissues through co-encapsulation (Cordero-Espinoza et al., 2021; Schindler et al., 2021), as exemplified with human PSC and mouse extraembryonic endoderm (XEN) cells (Schindler et al., 2021). Ultimately, this provides an important step for the establishment of scalable integrated stem cell-based embryo models to systematically interrogate lineage formation in primate embryogenesis.

## Supplementary Material

Movie 1

Movie 2

Supplementary file

## Data Availability

Single cell RNA-seq data for in vitro datasets have been deposited at ArrayExpress repository under accession number: E-MTAB-10639. In vivo reference data is available from previous publications, under accession E-MTAB-9367 Spatial Embryo Profiling of primate gastrulation. Processed data for analysis is available from: https://drive.google.com/file/d/13ElgKsZK1J1SESkwcNihWXV6kT-uR4Nz/view?usp=sharing Code is available from the GitHub repository: https://github.com/Boroviak-Lab/AmnionSpheroid
