# Supplementary material for: Microgel culture and spatial identity mapping elucidate the signalling requirements for primate epiblast and amnion formation
Source: Development. Author manuscript; Available in PMC 2023 Mar 25. (PMC7614365; doi:10.1242/dev.200263)
Supplement: Supplementary file [file EMS172352-supplement-Supplementary_file.pdf]

Supplementary figures

for the manuscript

Microgel culture and spatial identity mapping elucidate the signalling requirements for primate epiblast and amnion formation

Clara Munger[1,2,3,4#], Timo N. Kohler[3,4#], Erin Slatery[1,2,3#], Anna L. Ellermann[4], Sophie Bergmann[1,2,3], Christopher Penfold[1,2,3,5], Ioakeim Ampartzidis[1,2,3], Yutong Chen[1,2,3], Florian Hollfelder[4]* and Thorsten E. Boroviak[1,2,3,]*

Affiliations

1. Department of Physiology, Development and Neuroscience, University of Cambridge, Downing Site, Cambridge CB2 3EG, United Kingdom

2. Centre for Trophoblast Research, University of Cambridge, Downing Site, Cambridge CB2 3EG, United Kingdom

3. Wellcome Trust – Medical Research Council Stem Cell Institute, University of Cambridge, Jeffrey Cheah Biomedical Centre, Puddicombe Way, Cambridge CB2 0AW, United Kingdom

4. Department of Biochemistry, University of Cambridge, Hopkins Building, Tennis Court Road, Cambridge CB2 1QW, United Kingdom

5. Wellcome Trust – Cancer Research UK Gurdon Institute, Henry Wellcome Building of Cancer and Developmental Biology, University of Cambridge, Tennis Court Road, Cambridge, CB2 1QN, UK

* Correspondence: T.E.B. (teb45@cam.ac.uk), F.H. (fh111@cam.ac.uk)

[#] equal contribution

Fig. S1

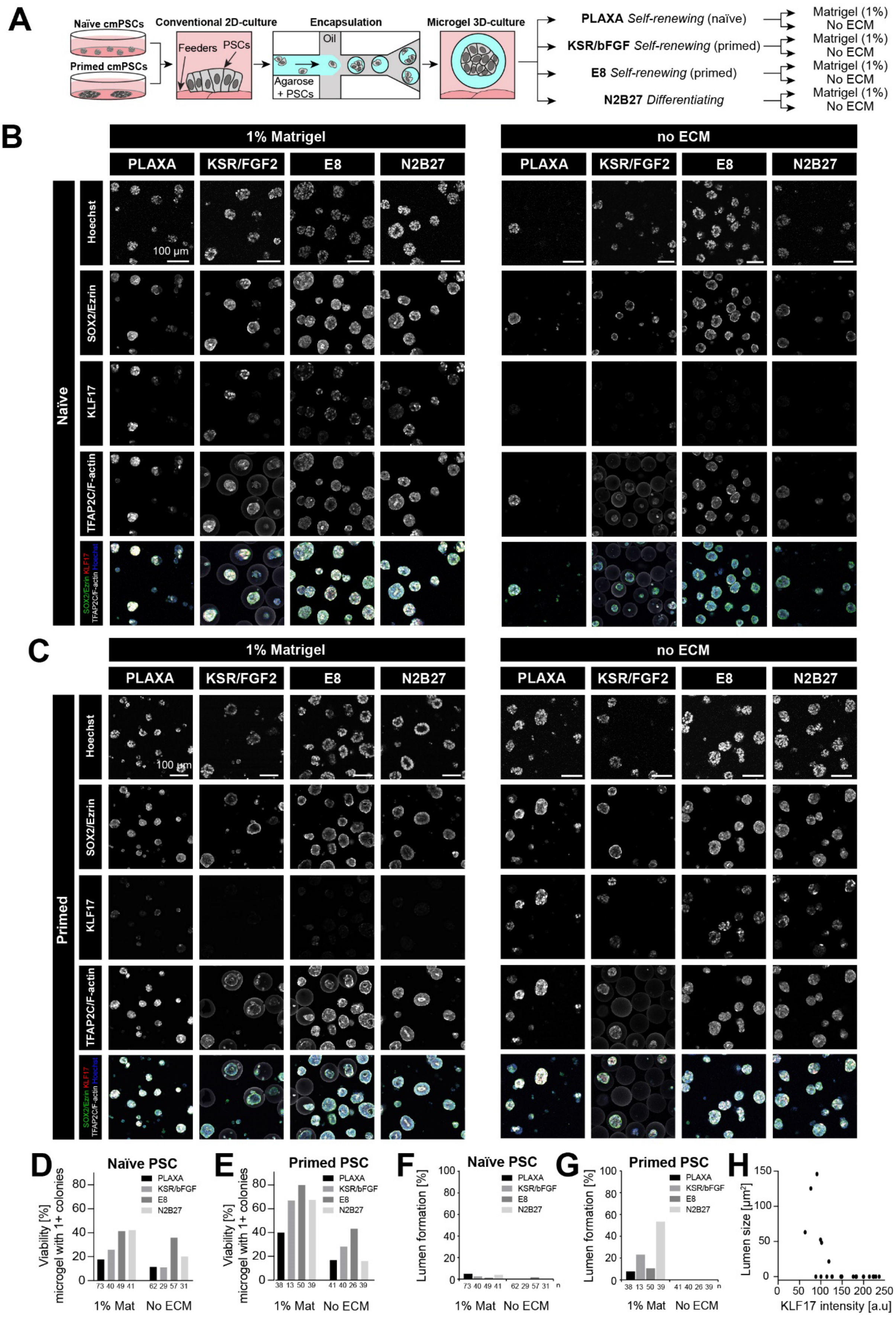
A
Naïve cmPSCs
Primed cmPSCs
Conventional 2D-culture
Feeders
PSCs
Encapsulation
Oil
Agarose + PSCs
Microgel 3D-culture
PLAXA Self-renewing (naïve)
KSR/bFGF Self-renewing (primed)
E8 Self-renewing (primed)
N2B27 Differentiating
Matrigel (1%)
No ECM
B
1% Matrigel
no ECM
PLAXA
KSR/FGF2
E8
N2B27
Naïve
Hoechst
SOX2/Ezrin
KLF17
TFAP2C/F-actin
100 µm
C
Primed
D
Naïve PSC
Viability [%] microgel with 1+ colonies
1% Mat
No ECM
E
Primed PSC
F
Lumen formation [%]
G
H
Lumen size [µm²]
KLF17 intensity [a.u]

Fig. S1 | Optimising marmoset microgel culture. A, Workflow for encapsulation of cmPSCs into agarose microgels and optimisation of culture conditions. **B**,**C**, Confocal immunofluorescence images of naïve (**B**) and primed (**C**) cmPSCs cultured in agarose microgels on MEFs and either supplemented with 1% Matrigel or without ECM in varying media compositions (PLAXA, KSR/FGF2, E8 and N2B27). **D**,**E**, Quantification of 3D-structures in agarose microgels after encapsulation of naïve (**D**) and primed (**E**) cmPSCs for the experimental conditions indicated. Number of structures quantified (n) indicated for each condition. (1% Mat: 1% Matrigel, ECM: extracellular matrix). **F**,**G**, Quantification of lumen formation of naïve (**F**) and primed (**G**) cmPSCs in microgels (number of structures quantified (n) indicated for each condition). **H**, Dynamics of lumen formation plotted against KLF17 expression in naïve cmPSCs cultured for 6 days in N2B27/1% Matrigel.

Fig. S2

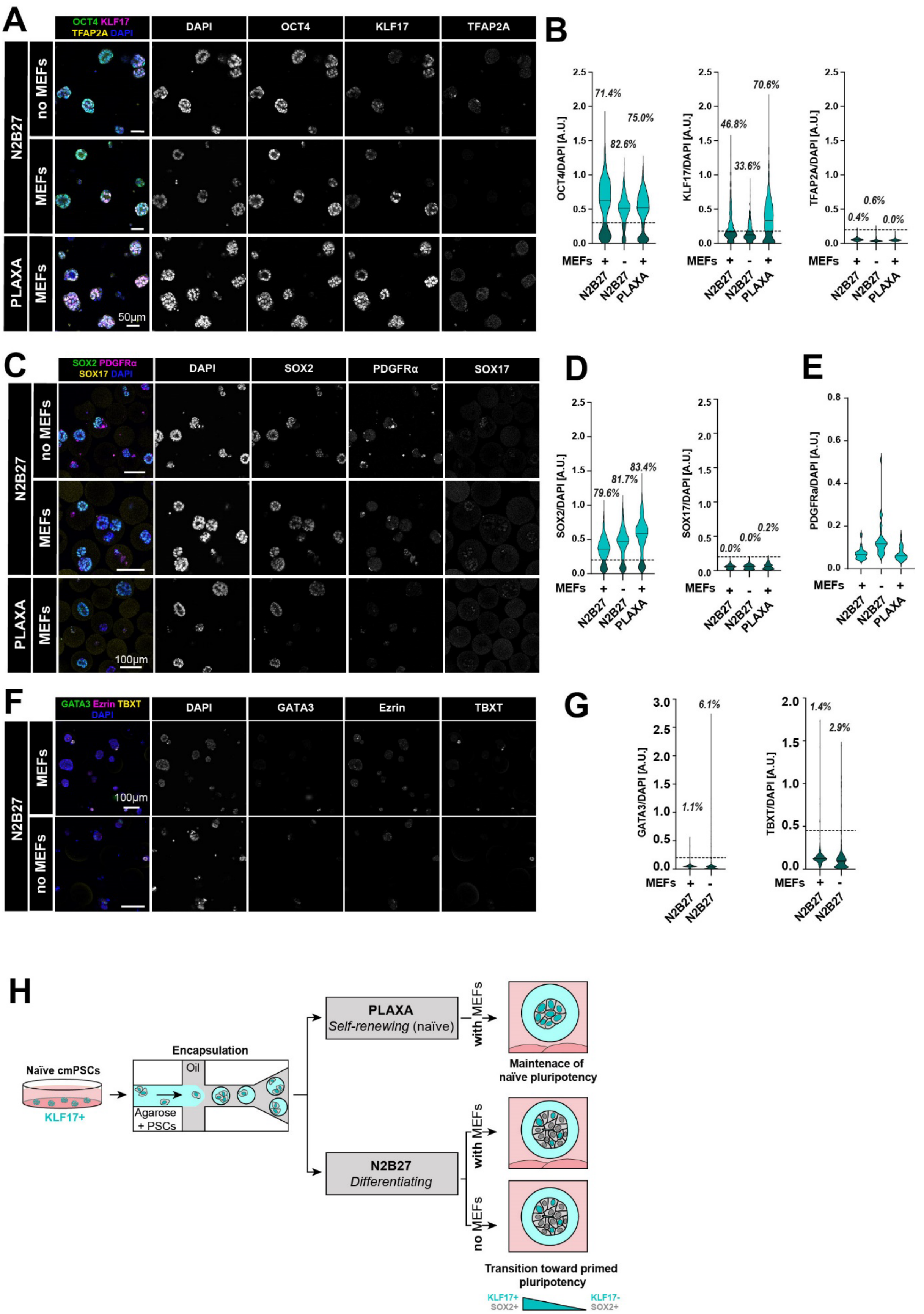
A
OCT4 KLF17 TFAP2A DAPI
DAPI
OCT4
KLF17
TFAP2A
N2B27
no MEFs
MEFs
PLAXA
50µm
B
OCT4/DAPI [A.U.]
71.4%
82.6%
75.0%
KLF17/DAPI [A.U.]
46.8%
33.6%
70.6%
TFAP2A/DAPI [A.U.]
0.4%
0.6%
0.0%
MEFs + - +
N2B27 N2B27 PLAXA
C
SOX2 PDGFRα SOX17 DAPI
SOX2
PDGFRα
SOX17
100µm
D
SOX2/DAPI [A.U.]
79.6%
81.7%
83.4%
SOX17/DAPI [A.U.]
0.0%
0.0%
0.2%
E
PDGFRa/DAPI [A.U.]
F
GATA3 Ezrin TBXT DAPI
GATA3
Ezrin
TBXT
G
GATA3/DAPI [A.U.]
1.1%
6.1%
TBXT/DAPI [A.U.]
1.4%
2.9%
MEFs + -
N2B27 N2B27
H
Naïve cmPSCs
KLF17+
Encapsulation
Oil
Agarose + PSCs
PLAXA
Self-renewing (naïve)
with MEFs
Maintenace of naïve pluripotency
N2B27
Differentiating
with MEFs
no MEFs
Transition toward primed pluripotency
KLF17+ SOX2+
KLF17- SOX2+

Fig. S2 | Lineage marker expression of microgel encapsulated naïve cmPSCs

A-G, Confocal immunofluorescence images of encapsulated naïve cmPSCs cultured in pluripotency (PLAXA with MEFs) or differentiation (N2B27 with MEFs and N2B27 no MEFs) conditions (**A**,**C**,**F**) and matched quantifications (**B,D,E,G**). Cells were stained for (**A**) OCT4, KLF17, TFAP2A, (**C**) SOX2, PDGFRα, SOX17 and (**F**) GATA3, Ezrin, TBXT and nuclei were counterstained with DAPI. Fluorescence intensity quantified on DAPI masks for one representative frame (**B,D,E,G**) or per structure on a sum of the slices of each z-stack (**E**). **H**, Schematic illustration of the cell fate of naïve cmPSCs encapsulated in agarose microgels cultured in various conditions.

Fig. S3

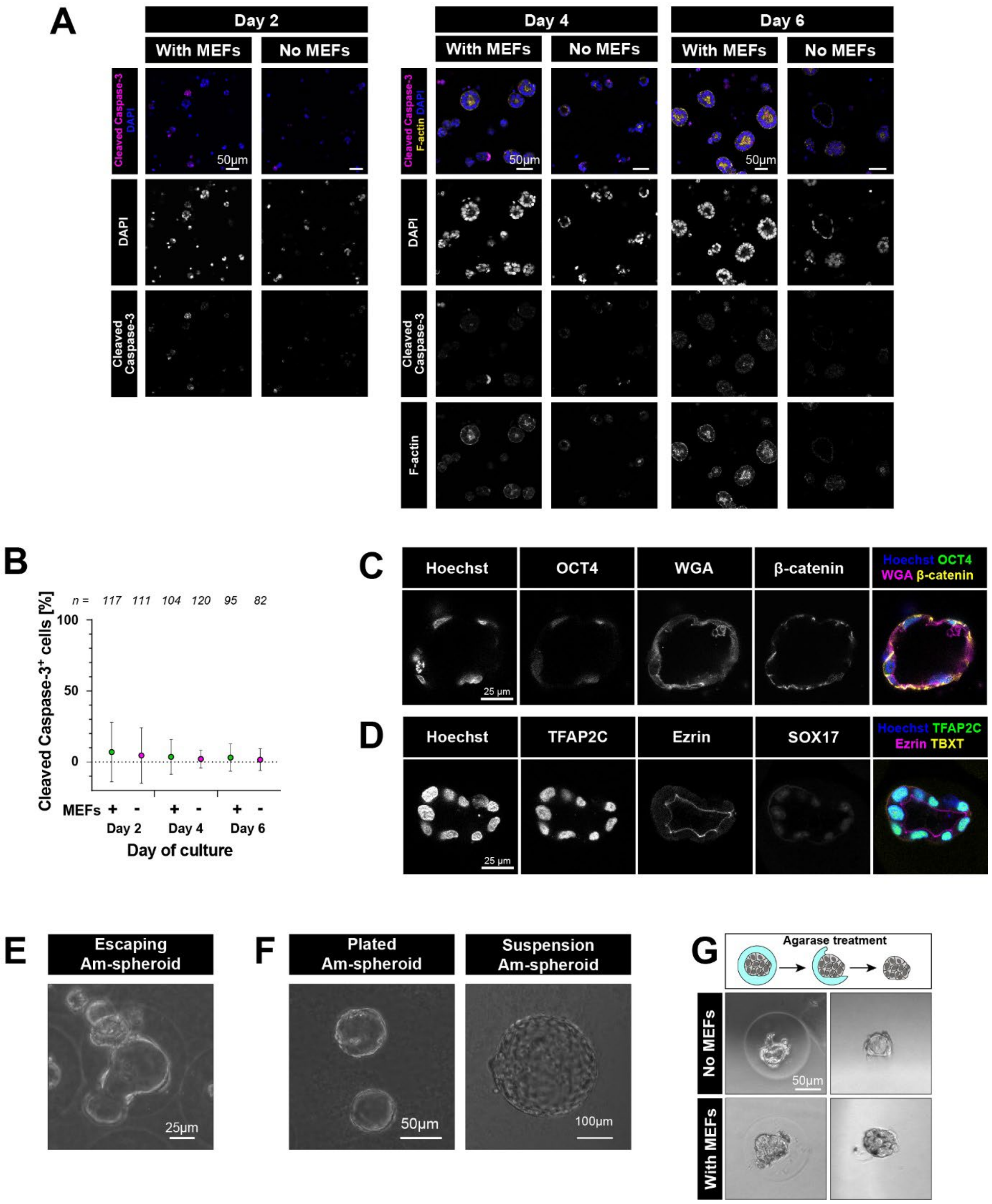

Fig. S3 | MEF removal leads to formation of Am-spheroids

A, Confocal images of encapsulated primed cmPSC cultured in N2B27 with and without MEFs and stained after 2, 4 and 6 days of culture. **B**, Weighted average of cells expressing cleaved

caspase-3 per structure. Number of structures quantified (n) indicated for each condition. **C,D**, Confocal images of Am-spheroids stained for. **E**, Phase contrast images of Am-spheroids in the process of escaping the agarose microgel. **F**, Phase contrast images of escaped Am-spheroids either grown on the bottom of the culture dish or cultured in suspension in a hanging drop at day 9. **G**, Phase contrast images of Am- and Epi-spheroids cultured in microgels before and after Agarase treatment.

Fig. S4

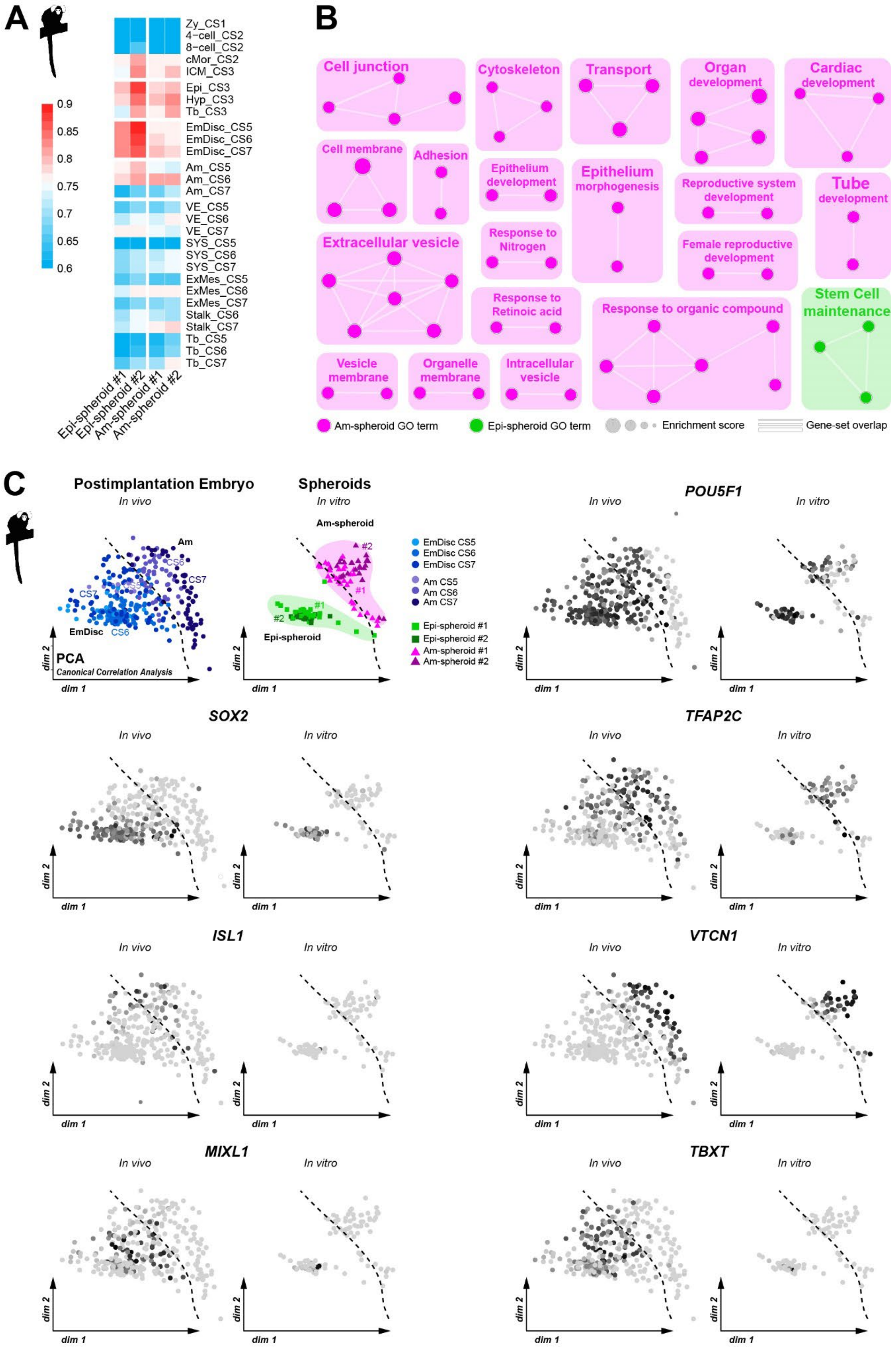
A
Zy_CS1
4−cell_CS2
8−cell_CS2
cMor_CS2
ICM_CS3
Epi_CS3
Hyp_CS3
Tb_CS3
EmDisc_CS5
EmDisc_CS6
EmDisc_CS7
Am_CS5
Am_CS6
Am_CS7
VE_CS5
VE_CS6
VE_CS7
SYS_CS5
SYS_CS6
SYS_CS7
ExMes_CS5
ExMes_CS6
ExMes_CS7
Stalk_CS6
Stalk_CS7
Tb_CS5
Tb_CS6
Tb_CS7
0.9
0.85
0.8
0.75
0.7
0.65
0.6
Epi-spheroid #1
Epi-spheroid #2
Am-spheroid #1
Am-spheroid #2
B
Cell junction
Cytoskeleton
Transport
Organ development
Cardiac development
Cell membrane
Adhesion
Epithelium development
Epithelium morphogenesis
Reproductive system development
Tube development
Extracellular vesicle
Response to Nitrogen
Female reproductive development
Response to Retinoic acid
Response to organic compound
Stem Cell maintenance
Vesicle membrane
Organelle membrane
Intracellular vesicle
Am-spheroid GO term
Epi-spheroid GO term
Enrichment score
Gene-set overlap
C
Postimplantation Embryo
In vivo
Spheroids
In vitro
Am-spheroid
Epi-spheroid
Am
EmDisc
CS5
CS6
CS7
#1
#2
EmDisc CS5
EmDisc CS6
EmDisc CS7
Am CS5
Am CS6
Am CS7
Epi-spheroid #1
Epi-spheroid #2
Am-spheroid #1
Am-spheroid #2
PCA
Canonical Correlation Analysis
dim 1
dim 2
POU5F1
SOX2
TFAP2C
ISL1
VTCN1
MIXL1
TBXT
In vivo
In vitro

Fig. S4 | Genome-wide transcriptional comparison of Epi- and Am-spheroids to the marmoset embryo

A, Pearson correlation analysis of Epi- and Am-spheroids to pre- and postimplantation lineages of the marmoset embryo *in vivo*. **B**, Gene ontology for Am-spheroid (pink) and Epi-spheroid (green) specific genes. **C**, Principal component analysis of adjusted expression values based on a set of genes identified via canonical correlation analysis. Displayed are the EmDisc (CS5-7), amnion (CS5-7) and Epi- and Am-spheroids. Absolute gene expression levels are displayed for the genes indicated.

Fig. S5

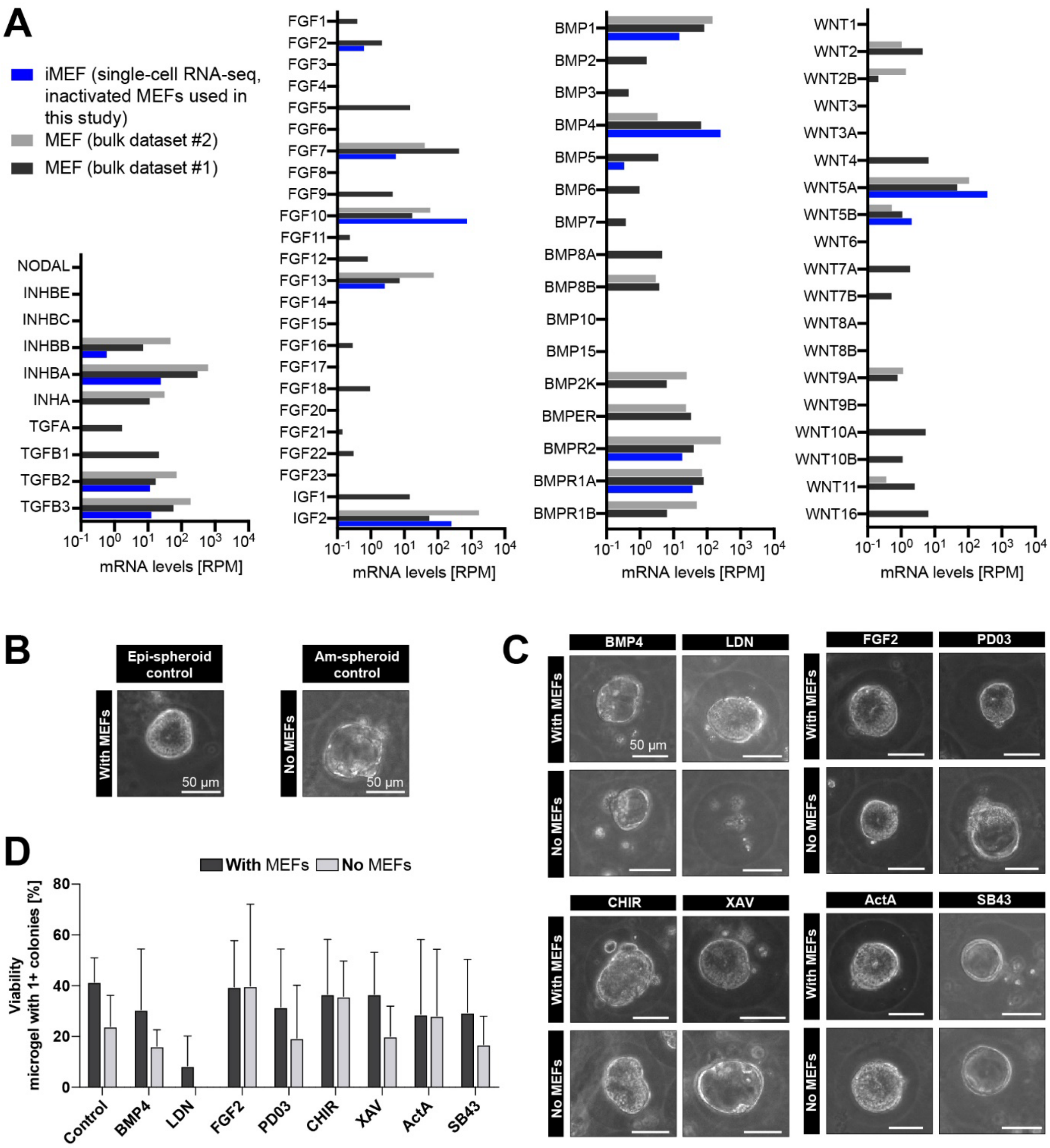

Fig. S5 | Lineage conversion of spheroids in Epi- and Am-culture conditions upon signalling pathway modulation

A, Ligand expression of MEFs. Gene expression in reads per million (RPM) of selected ligands of the FGF/MAPK, BMP, WNT and NODAL/TGFβ signalling pathways. **B**, Phase contrast images of control Epi- and Am-spheroids. **C**, Phase contrast images of Epi- and Am-

spheroids under tested conditions. **D**, Structure formation (microgel containing at least one colony) of microgel cultured primed cmPSCs (N=2, two independent cell lines).

Fig. S6

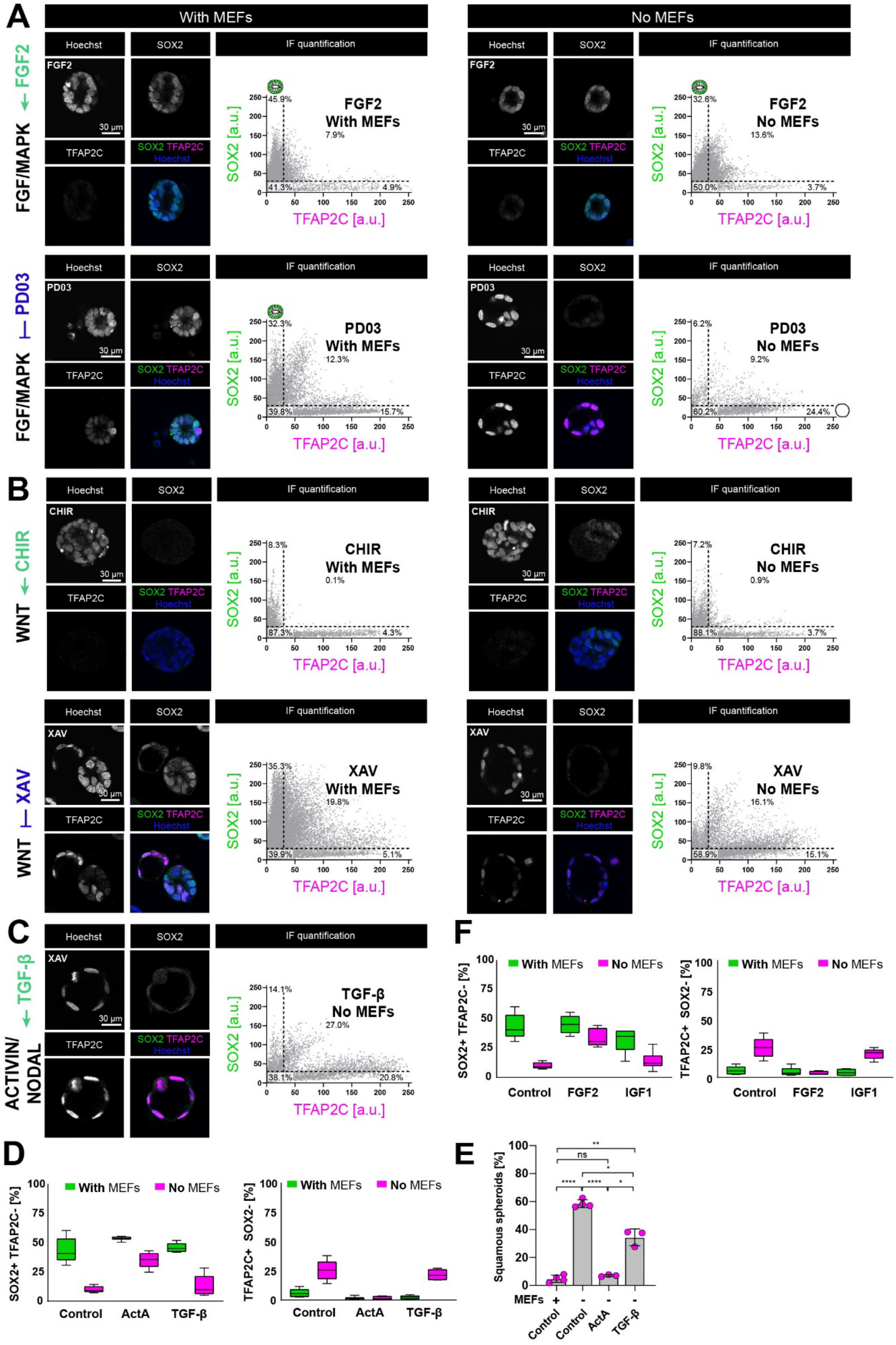

A
With MEFs
No MEFs
Hoechst
SOX2
TFAP2C
IF quantification
FGF/MAPK → FGF2
FGF2 With MEFs
FGF2 No MEFs
FGF/MAPK ⊣ PD03
PD03 With MEFs
PD03 No MEFs
B
WNT → CHIR
CHIR With MEFs
CHIR No MEFs
WNT ⊣ XAV
XAV With MEFs
XAV No MEFs
C
ACTIVIN/NODAL → TGF-β
TGF-β No MEFs
SOX2 [a.u.]
TFAP2C [a.u.]
30 µm
D
SOX2+ TFAP2C- [%]
TFAP2C+ SOX2- [%]
Control
ActA
TGF-β
E
Squamous spheroids [%]
MEFs
F
FGF2
IGF1

Fig. S6 | Quantitative assessment of lineage marker expression upon single pathway perturbation

A,**B**, Confocal immunofluorescence of Epi- and Am-spheroids treated with (**A**) FGF2 and PD03 and (**B**) CHIR and XAV. Fluorescence intensity (shown on the right) was quantified on Hoechst masks for each frame in the z-stack. **C**,**D**, Percentage of SOX2+, TFAP2C- and SOX2-, TFAP2C+ nuclear per condition for the experimental conditions indicated. Boxplots represent variability across z-stacks. **E**, Squamous spheroid forming capacity of microgel cultured primed cmPSCs at day 6 (N=3 independent experiments). Comparisons conducted using two-tailed t-test with Welch's correction: Control with MEFs vs. no MEFs: p<0.0001, Control with MEFs vs. TGF-b: p=0.0068, Control no MEFS vs. ActA: p<0.0001, Control no MEFs vs. TGF-□: p=0.0105, ActA vs. TGF-□: p=0.0143). **F,** Percentage of SOX2+, TFAP2C- and SOX2-, TFAP2C+ nuclear per condition for the experimental conditions indicated. Boxplots represent variability across z-stacks.

Fig. S7

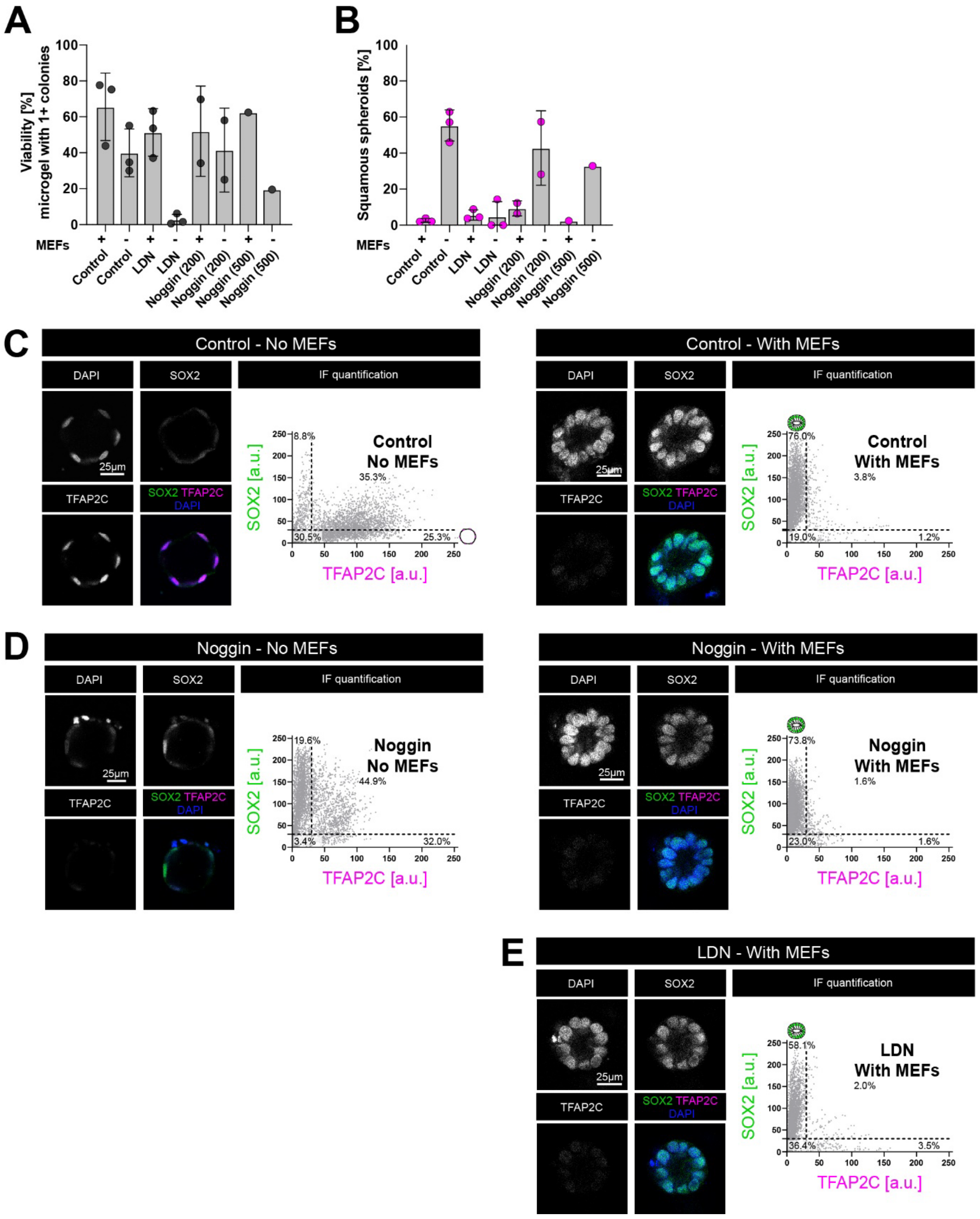

Fig. S7 | BMP inhibition in amnion specification

A, Structure formation (microgels containing at least one colony) of encapsulated cmPSCs under tested conditions: Epi- and Am-spheroid (control, N=3), LDN at a concentration of 500

nM (LDN, N=3), Noggin at a concentration of 200 ng/ml (Noggin (200), N=2) and of 500 ng/ml (Noggin (500), N=1). **B,** Squamous spheroid forming capacity of encapsulated cmPSCs at day 6. Every condition was tested with and without MEFs: Control N=3, LDN N=3, Noggin (200) N=2, Noggin (500) N=1. **C**-**E**, Confocal immunofluorescence images of Epi- and Am-spheroids treated with (**c**) Noggin at 200 ng/ml and (**D**) LDN. Fluorescence intensity was quantified on DAPI masks for each frame in the z-stack.

Fig. S8

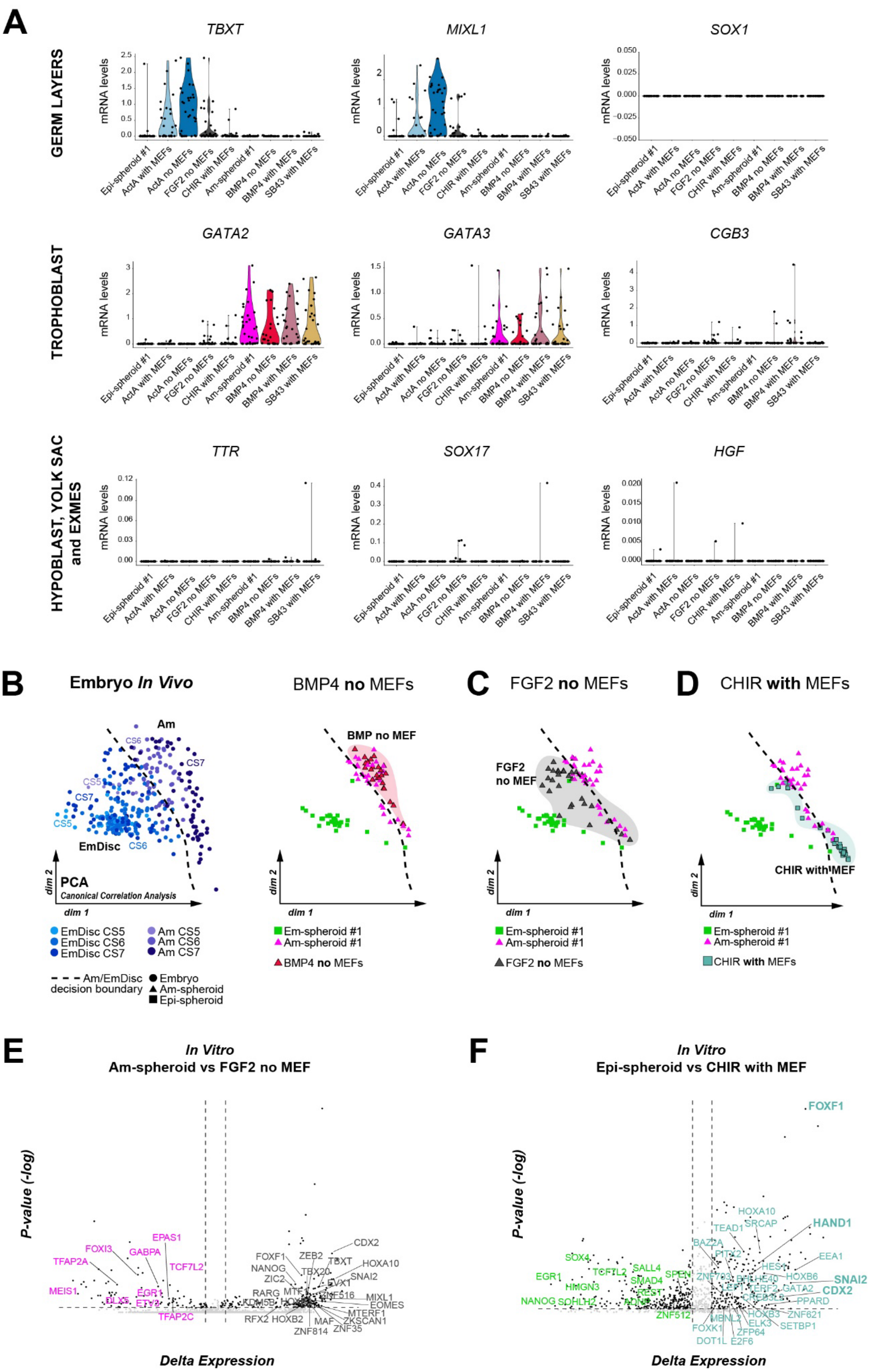

A
GERM LAYERS
TBXT
MIXL1
SOX1
TROPHOBLAST
GATA2
GATA3
CGB3
HYPOBLAST, YOLK SAC and EXMES
TTR
SOX17
HGF
mRNA levels
Epi-spheroid #1
ActA with MEFs
ActA no MEFs
FGF2 no MEFs
CHIR with MEFs
Am-spheroid #1
BMP4 no MEFs
BMP4 with MEFs
SB43 with MEFs
B
Embryo In Vivo
BMP4 no MEFs
C
FGF2 no MEFs
D
CHIR with MEFs
PCA
Canonical Correlation Analysis
dim 1
dim 2
EmDisc CS5
EmDisc CS6
EmDisc CS7
Am CS5
Am CS6
Am CS7
Am/EmDisc decision boundary
Embryo
Am-spheroid
Epi-spheroid
Em-spheroid #1
Am-spheroid #1
BMP4 no MEFs
FGF2 no MEFs
CHIR with MEFs
E
In Vitro
Am-spheroid vs FGF2 no MEF
F
In Vitro
Epi-spheroid vs CHIR with MEF
P-value (-log)
Delta Expression

Fig. S8 | Absence of germ layer and extraembryonic lineage marker expression in Epi- and Am-spheroids

A, Violin plots showing lineage marker mRNA expression of control Epi- and Am-spheroids and lineage converted structures obtained by single pathway modulation. **B**-**D**, Principal component analysis of adjusted expression values based on a set of genes identified via canonical correlation analysis comparing control Epi- and Am-spheroids with samples by single pathway modulation: FGF2 without MEFs (**B**), BMP4 without MEFs (**C**), and CHIR with MEFs (**D**). Silhouettes highlight the perturbation shown in each plot. **E-F,** Differentially expressed genes in the (**E**) Am-spheroid control *vs*. FGF2 without MEFs and (**F**) Epi-spheroid control *vs*. CHIR with MEFs. Transcription factor gene names are displayed with advanced mesoderm markers in bold.

Fig. S9

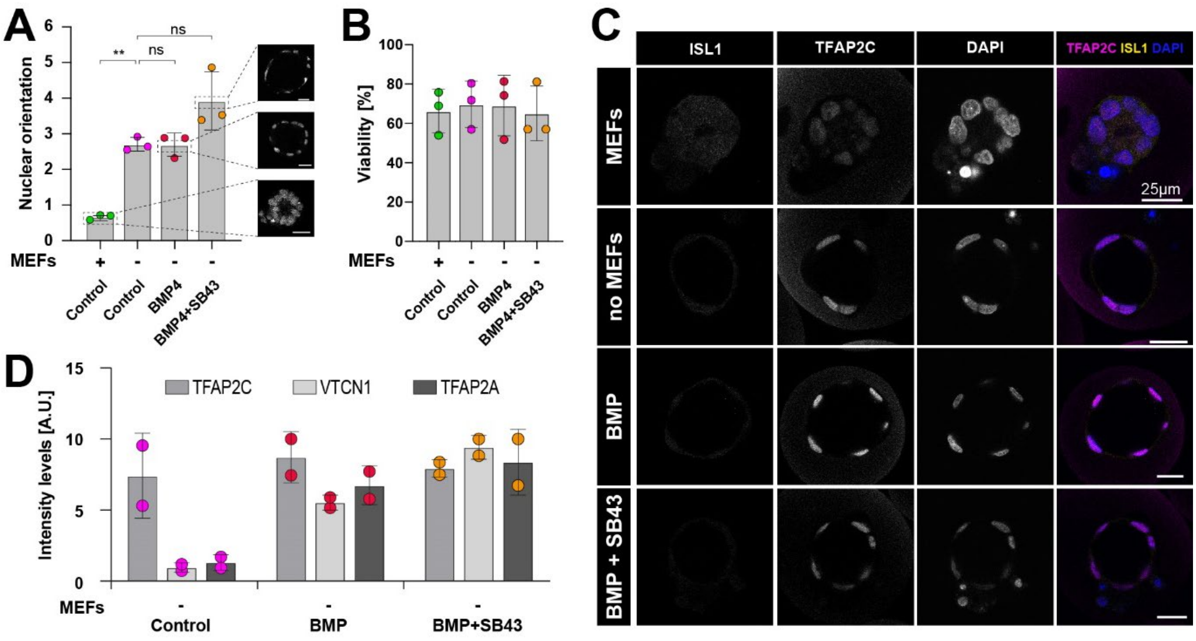

Fig. S9 | Assessment of combinatorial signalling on Am-spheroid formation

A, Quantification of nuclear orientation for the experimental conditions indicated (N=3). Representative confocal images are shown on the right (scale bar: 25 µm). Comparisons conducted using two-tailed t-test with Welch's correction (Control with MEF vs. Control no MEF: p=0.001, Control no MEF vs. BMP: p=0.9613, Control no MEF vs. BMP+SB43: p=0.1158). **B,** Structure formation (microgels containing at least one colony) of microgel cultured primed cmPSCs under tested conditions (N=3). **C,** Confocal immunofluorescence images of control Am- and Epi-spheroid and of Am-spheroids under the indicated conditions at day 6. **D,** Fluorescence intensities of the confocal images quantified on DAPI masks for one representative frame (TFAP2C and TFAP2A) or per structure on a sum of the slices of each z-stack (VTCN1). Intensities were first normalised by the DAPI signal then averages were divided by the average of the Epi-spheroid condition. Related to Fig. 7e.

Supplementary Movies

Movie 1 | Confocal wholemount immunofluorescence staining of an Epi-spheroid for DAPI (blue), SOX2 (green), PARD6 (red) and TFAP2C (white).

Movie 2 | Confocal wholemount immunofluorescence staining of an Am-spheroid for DAPI (blue), SOX2 (green), PARD6 (red) and TFAP2C (white).
